# An *Nlrp5*-null mutation leads to attenuated *de novo* methylation in oocytes, accompanied by a significant reduction in DNMT3L

**DOI:** 10.1093/molehr/gaaf055

**Published:** 2025-11-18

**Authors:** Leah Nic Aodha, Alexandra Pokhilko, Leah U Rosen, Styliani Galatidou, Edyta Walewska, Christian Belton, Antonio Galvao, Hanneke Okkenhaug, Lu Yu, Asif Nakhuda, Bill Mansfield, Soumen Khan, David Oxley, Montserrat Barragán, Gavin Kelsey

**Affiliations:** Epigenetics Programme, Babraham Institute, Cambridge, UK; Hughes Hall, University of Cambridge, Cambridge, UK; Epigenetics Programme, Babraham Institute, Cambridge, UK; Darwin College, University of Cambridge, Cambridge, UK; Research Department, European Bioinformatics Institute, Hinxton, UK; Research and Development, EUGIN Group, Barcelona, Spain; School of Agriculture and Food Science, University College Dublin, Dublin, Ireland; Epigenetics Programme, Babraham Institute, Cambridge, UK; Epigenetics Programme, Babraham Institute, Cambridge, UK; Loke Centre for Trophoblast Research, University of Cambridge, Cambridge, UK; Royal Veterinary College, University of London, London, UK; Imaging Facility, Babraham Institute, Cambridge, UK; Mass-spectrometry Facility, Babraham Institute, Cambridge, UK; Gene Targeting Facility, Babraham Institute, Cambridge, UK; Biological Support Unit, Babraham Institute, Cambridge, UK; Epigenetics Programme, Babraham Institute, Cambridge, UK; Mass-spectrometry Facility, Babraham Institute, Cambridge, UK; Research and Development, EUGIN Group, Barcelona, Spain; Epigenetics Programme, Babraham Institute, Cambridge, UK; Loke Centre for Trophoblast Research, University of Cambridge, Cambridge, UK; Wellcome-MRC Institute of Metabolic Science-Metabolic Research Laboratories, University of Cambridge, Cambridge, UK

**Keywords:** oocyte, epigenetics, imprinting, DNA methylation, multi-omics, *Nlrp5*, subcortical maternal complex

## Abstract

*Nlrp5* encodes a core component of the subcortical maternal complex (SCMC), a cytoplasmic protein structure unique to the mammalian oocyte and cleavage-stage embryo. *NLRP5* mutations have been identified in patients presenting with early embryo arrest, recurrent molar pregnancies, and imprinting disorders. Correct patterning of DNA methylation over imprinted domains during oogenesis is necessary for faithful imprinting of genes. It was previously shown that oocytes with mutation in the human SCMC gene *KHDC3L* had globally impaired methylation, indicating that integrity of the SCMC is essential for correct establishment of DNA methylation at imprinted regions. Here, we present a multi-omic analysis of an *Nlrp5*-null mouse model, which in germinal vesicle (GV)-stage oocytes displays a misregulation of a broad range of maternal proteins, including proteins involved in several key developmental processes. This misregulation likely underlies impaired oocyte developmental competence. Amongst impacted proteins are several epigenetic modifiers, including a substantial reduction in DNMT3L; we show that *de novo* DNA methylation is attenuated in *Nlrp5*-null oocytes, including at some imprinting control regions. This provides evidence for a mechanism of epigenetic impairment in oocytes, which could contribute to downstream misregulation of imprinted genes.

## Introduction

The mammalian oocyte provides not only the maternal genetic contribution to the embryonic genome, but also the proteins and mRNAs necessary to sustain the newly fertilized zygote through the first stages of embryogenesis, until zygotic genome activation and beyond (Zuccotti *et al.*, 2011; Yeste *et al.*, 2017; Cheng *et al.*, 2022; Jentoft *et al.*, 2023). During primordial germ cell specification, the epigenetic landscape is erased and re-established after sex specification, in order to restore gametic pluripotency. As the growing oocyte develops, DNA methylation patterning is re-established in a transcription-dependent manner, and is completed upon reaching the surrounded nucleolus (SN) stage of nuclear maturation (Demond *et al.*, 2024), at which point the oocyte becomes largely transcriptionally silent (Chotalia *et al.*, 2009; Nikpour *et al.*, 2009; Smallwood and Kelsey, 2012; Demond and Kelsey, 2020). The DNA methylation landscape in the oocyte differs significantly from that of somatic cells. While considered a repressive modification in somatic cells, DNA methylation in oocytes does not coincide with silenced gene regulatory elements. Instead, DNA methylation is present in a bimodal pattern of hypermethylated but actively transcribed regions, and hypomethylated transcriptionally inactive regions (Demond and Kelsey, 2020). Later stages of oocyte maturation are orchestrated by its pre-existing pool of mRNAs and proteins (Li *et al.*, 2010). Upon fertilization, the newly created zygote undergoes another wave of global DNA methylation erasure, with the exception of methylation at imprinted regions, which is retained to ensure parent-of-origin gene expression (Bartolomei and Ferguson-Smith, 2011; Smallwood and Kelsey, 2012).

While the establishment of DNA methylation in the mouse oocyte is catalysed by DNA methyltransferase DNMT3A and requires its cofactor DNMT3L (Smallwood *et al.*, 2011; Uysal *et al.*, 2017), the maintenance of methylation through cell divisions post-fertilization is dependent on DNMT1, regulated by accessory proteins UHRF1 and DPPA3 (Sharif *et al.*, 2007; Maenohara *et al.*, 2017; Kiefer and Simon, 2019; Lin *et al.*, 2023). DNMT1 also maintains parent-of-origin-specific methylation at imprinted regions during global methylation erasure events (Hirasawa *et al.*, 2008). Maintenance post-fertilization of methylation specifically at imprinted regions also requires the KRAB zinc finger proteins ZFP57 and ZNF445 together with TRIM28 (KAP1, TIF1B) (Messerschmidt *et al.*, 2012; Juan and Bartolomei, 2019). Both UHRF1 and DNMT1 can also contribute to *de novo* methylation in oocytes (Shirane *et al.*, 2013; Maenohara *et al.*, 2017; Cao *et al.*, 2019), and UHRF1 has been shown to play other important roles in the oocyte, such as protecting against DNA damage (Cao *et al.*, 2019) and regulating the cytoplasmic architecture and function in the oocyte via tubulins and other microtubule-related proteins (Uemura *et al.*, 2023).

However, UHRF1 and DNMT1 are considered particularly crucial for maintenance of DNA methylation. DNMT3A and DNMT3L are predominantly localized to the nucleus in oocytes and cleavage-stage embryos, whereas DNMT1 and its accessory protein UHRF1 are predominantly localized to the cytoplasm during this developmental window (Maenohara *et al.*, 2017), although some DNMT1/UHRF1 is retained in the nucleus (Mertineit *et al.*, 1998; Hirasawa *et al.*, 2008), indicating an important role of regulated sub-cellular localization in controlling DNA methylation dynamics. Given that later stages of oogenesis and early stages of embryogenesis are directed by the oocyte pool of proteins and mRNAs, investigating the dynamics of the oocyte proteome may provide clues to understanding the mechanisms by which DNA methylation establishment and maintenance are regulated, with important implications for the understanding of DNA methylation-related disorders, such as multi-locus imprinting disturbances (MLIDs) and imprinting syndromes in humans (Riccio *et al.*, 2019).

The subcortical maternal complex (SCMC) is an enigmatic protein complex unique to the mammalian oocyte and cleavage-stage embryo. This complex is composed of several of the most highly abundant oocyte proteins, encoded by maternal-effect genes, although its function remains to be fully characterized (Ohsugi *et al.*, 2008; Lu *et al.*, 2017; Bebbere *et al.*, 2021). Structures of the core complex, comprising NALP5 (or NLRP5, MATER), OOEP (FLOPED), TLE6, and KHDC3 (FILIA), in both human and mouse, have recently been solved (Chi *et al.*, 2024a, 2024b). Recent studies on SCMC proteins have focused on their relationship with cytoplasmic lattices (CPLs), another previously described oocyte structure with incomplete characterization of function. CPLs are found throughout the cytoplasm, and are likely composed of the core SCMC and associated proteins, including PADI6, suggesting that SCMC proteins are not in fact subcortical in nature (Kim *et al.*, 2010; Tashiro *et al.*, 2010; Morency *et al.*, 2011; Jentoft *et al.*, 2023). In light of this new framing, SCMC–CPL structures are thought to be involved in sequestration and protection of important developmental proteins from premature degradation (Yurttas *et al.*, 2008; Tashiro *et al.*, 2010; Jentoft *et al.*, 2023). Interestingly, mutations in SCMC genes have been associated with aberrantly high DNA methylation in murine pre-implantation embryos, in some cases coupled with altered localization of DNMT1 and UHRF1 (Zhang *et al.*, 2023; Giaccari *et al.*, 2024). Mutant variants of SCMC genes such as *KHDC3L*, *PADI6*, and *NLRP5* have been identified in patients presenting with recurrent biparental complete hydatidiform moles (Demond *et al.*, 2019), and offspring with MLIDs and imprinting disorders (Eggermann *et al.*, 2021; Rezaei *et al.*, 2021), suggesting a link between the SCMC and either establishment or retention of DNA methylation patterning at imprinted genes. Furthermore, human oocytes with inactivating mutations in *KHDC3L* are globally hypomethylated (Demond *et al.*, 2019). The difficulty in determining a causal link between SCMC variants and downstream disordered DNA methylation lies in the diversity of clinical outcomes of SCMC mutations in humans, coupled with limited human material for analysis, and the fact that SCMC-null mutations in mice usually lead to embryonic arrest prior to zygotic genome activation (Yurttas *et al.*, 2008; Fernandes *et al.*, 2012; Alazami *et al.*, 2015; Qin *et al.*, 2019; Zheng *et al.*, 2021). Even when human SCMC-related imprinting disorder variants are mimicked in the murine system, they have been shown to lead to two-cell arrest (Giaccari *et al.*, 2024). Nonetheless, the tractability of mouse knockouts enables them to be a valuable tool in analysing the consequences of inactivating SCMC mutations, despite potential imperfections.

We focus on understanding the potential causes of developmental incompetence in oocytes using a novel mutation in the core SCMC gene and known maternal-effect gene *Nlrp5.* To understand how disruption of the SCMC in oocytes might lead to disordered DNA methylation and adverse developmental outcomes, we employ a multi-omic approach, profiling the oocyte proteome, transcriptome, and DNA methylation landscape, in addition to investigating the localization of key epigenetic modifier proteins in *Nlrp5*^−/−^ oocytes.

## Materials and methods

### Experimental model and subject details

All animal experimental procedures were approved by the Animal Welfare and Ethical Review Body at the Babraham Institute and were conducted under authority of the UK Home Office issued licences in accordance with the Animal (Scientific Procedures) Act 1986.

### Generation and verification of the *Nlrp5* knockout mouse line

The *Nlrp5* knockout (KO) mouse model was generated using CRISPR/Cas9 technology by directly targeting mouse zygotes. Specifically, a single guide RNA (sgRNA) was designed using Benchling (https://www.benchling.com/crispr), and the guide sequence TGTCGAACTTAGCAATCACA was identified within a constitutive exon and essential protein domain. The CRISPR editing was performed by the Babraham Institute Gene Targeting Facility. Mouse zygotes were obtained from superovulated C57BL6/Babr females, and Cas9 protein (100 ng/µl, IDT, Coralville, IA, USA) along with sgRNA (100 ng/µl, IDT, Coralville, IA, USA) diluted in Opti-MEM (Thermo Fisher Scientific, Waltham, MA, USA) was introduced into the zygotes via electroporation using the NEPA21 device (Nepa Gene, Ichikawa City, Japan) with the following settings: 40 V, 3.5 ms pulse lengths, 50 ms intervals, and 4 pulses. The embryos were cultured overnight, and on the following day, two-cell stage embryos were transferred into pseudopregnant CD1 females. Ear clips were collected from the F0 pups post-weaning for genotyping to identify KO genotypes. Genotyping PCR amplification was conducted over the Cas9/sgRNA-induced double-strand break using the primers: forward 5′-CCATTCAGGTTTCCTCCCAT-3′ and reverse 5′-CCTGGTCTTCCATGTAGGAT-3′ (IDT, Leuven, Belgium). Samples were purified using the Zymo DNA extraction kit (Zymo Research, Irvine, CA, USA) and sent for Sanger sequencing (Azenta, Takeley, UK). Sanger sequencing traces were analysed using Inference of CRISPR Edits (ICE; Synthego, Redwood, CA, USA) to identify F0 mice with KO genotypes. These F0 mice were then crossed with wild-type C57BL6/Babr females to produce F1 offspring. Homozygous and heterozygous mutant mice for the collection of oocytes were obtained at the F2 and subsequent generations. Genotyping was performed by Transnetyx (Memphis, TN, USA) using a TaqMan-based assay to obtain real-time PCR data for automated detection of the desired frameshift mutation. Subsequent litters were also genotyped via Transnetyx using the same TaqMan-based assay.

### Timed matings and animal husbandry (litter counts/sizes)

All mice used in this study were bred and maintained in the Babraham Institute Biological Support Unit (BSU). Ambient temperature was ∼19–21°C and relative humidity was 52%. Lighting was provided on a 12-h light: 12-h dark cycle, including 15 min ‘dawn’ and ‘dusk’ periods of subdued lighting. After weaning, mice were transferred to individually ventilated cages with 1–5 mice per cage. Mice were fed CRM (P) VP diet (Special Diet Services, Witham, UK) *ad libitum* and received seeds (e.g. sunflower, millet) at the time of cage-cleaning as part of their environmental enrichment. Animal husbandry was managed by the BSU, including the setting up of timed matings and routine genotyping of young mice (Transnetyx), once the *Nlrp5* KO colony had been established.

### Oocyte and embryo collections

Female mice were sacrificed at 3 weeks old, and ovaries were dissected out and collected in M2 medium (Merck, Lebanon, NJ, USA) on ice. Sacrificed *Nlrp5*^−/−^ and *Nlrp5^+/^*^−^ females were always littermates, and matched *Nlrp5*^+/+^ females were usually from the originating C57BL6/Babr colony. For immunofluorescence and proteomics analysis, germinal vesicle (GV)-stage oocytes were isolated from ovaries in M2 medium using the scratch method, whereby oocytes were released from the ovary by gently scratching the ovary with a fine needle. No hormonal stimulation was employed prior to oocyte collection. Oocytes were manually washed in 4–5 drops of M2 medium using a drawn-out mouth pipette. Oocytes were then rinsed in phosphate-buffered saline (PBS, prepared in-house) 3 times to minimize bovine serum albumin (from the M2 medium) contamination during mass spectrometry analysis. They were then either fixed in 4% paraformaldehyde (PFA; Merck, Lebanon, NJ, USA) for 15 min for downstream immunostaining and stored in PBS–Tween 20 (referred to as ‘PBST’: PBS with 0.05% Tween 20, Sigma-Aldrich, St Louis, MO, USA) at 4°C, or snap frozen in 5 µl PBS for mass spectrometry analysis. For single-cell methylation and transcriptome sequencing, oocytes were instead collected using a collagenase digestion method (to reduce chance of somatic cell contamination). The digestion mix was prepared as follows, for each pair of ovaries: 454.2 µl dPBS (with Mg^2+^, Ca^2+^; Thermo Fisher Scientific), 33.3 µl 30 mg/ml Collagenase (Merck, Lebanon, NJ, USA), 12.5 µl 1% Trypsin (Merck, Lebanon, NJ, USA). For the collagenase digest method, ovaries were dissected out of young mice (21–25 days old) and rinsed in PBS. A small nick was made in each ovary using a fine needle. Each pair of ovaries were added to a vial of digestion mix and incubated at 37°C with constant agitation (550 rpm) for 25 min. After 5 min, the ovaries were pipetted up and down 5 times with a P1000 pipette, set to 500 µl. After 10 min, the ovaries were pipetted gently, by up-taking the whole tissue with a P200 pipette, set to 200 µl, around 40 times. After 10 min, the ovaries were pipetted 10 times with the P200 pipette, disrupting any clumps that remained in the ovary. The digestion mix was then transferred to a 100 mm coated dish (Nunc; Merck, Lebanon, NJ, USA) and checked for viscosity under a dissection microscope. When most oocytes were free from somatic cells, and viscosity was not too high, 500 µl of M2 medium was added to the digestion mix to stop the digest. GV-stage oocytes were isolated from the mixture and washed through 4–5 droplets of M2 medium using a mouth pipette. Oocytes were rinsed once in PBS, and snap frozen on dry ice in 2–5 µl RLT+ buffer (QIAGEN, Hilden, Germany) in low-bind 0.2 ml PCR strip tubes (Merck, Lebanon, NJ, USA).

Embryo collections from mated females were performed during KO characterization to determine the fertility of different genotype combinations in the *Nlrp5* mutant colony, and to investigate whether this KO recapitulated the two-cell arrest phenotype *in vivo*. Mated females were plug-checked to estimate the timing of fertilization, and oviducts and uteruses were dissected out at E3.5. Tissues were cleaned in M2 medium using tweezers and fine needles, trimming away fat. A syringe was filled with M2 medium and inserted into one end of the oviduct, all within a large droplet of M2 medium. The liquid was expelled from the syringe gently, rinsing any embryos in the oviduct out into a glass collection dish. Embryos were gently rinsed in PBS using a mouth pipette and visually staged under a dissection microscope.

### Immunofluorescence staining of oocytes and embryos

Fixed oocytes or embryos were washed in PBST, permeabilized in PBS 0.5% Triton X-100 (Thermo Fisher Scientific) for 1 h at room temperature, and then washed again in PBST. For Triton X-100 extraction (Kim *et al.*, 2010), unfixed oocytes were incubated in extraction buffer containing 0.1 M KCl, 20 mM MgCl_2_, 3 mM EGTA, 20 mM HEPES (pH 6.8), 0.1% Triton X-100 and 1× Complete Protease Inhibitor Cocktail (Roche, Basel, Switzerland) for 10 min and rinsed in PBS quickly and fixed and permeabilized as above. Samples were blocked for 1 h at room temperature, in PBS (0.05%)–Tween-20 (1%) bovine serum albumin (BSA) solution (referred to as BS) (BSA from Merck, Lebanon, NJ, USA), and then incubated for 1 h at room temperature with the appropriate dilution of the primary antibody in BS (NALP5 rabbit polyclonal antibody, gifted by Jurrien Dean, NIH, USA, 1:50. DNMT1, Abcam, Cambridge, UK, cat: AB87654, 1:500. UHRF1, MBL Lifescience, Japan, cat: D289-3, 1:100. DNMT3L, gifted by Shoji Tajima, Osaka University, Japan, 1:100. α-tubulin, Thermo Fisher Scientific, cat.: 236-10501, 1:100. γ-tubulin, Thermo Fisher Scientific, cat: MA1-19421, 1:100. H3 cat: MAB3422 Anti-Histone Antibody, clone H11-4, 1:100, Thermo Fisher Scientific). Samples were then washed in BS for at least 1 h at room temperature, and then incubated with the appropriate secondary antibody (AlexaFluor 488, 561 or 647, 1:500, Thermo Fisher Scientific) and two drops of Hoechst reagent (NucBlue Live Ready Probes, 33342, Thermo Fisher Scientific, cat: R37605) in 500 µl BS for 1 h at room temperature in the dark. Samples were washed for 1 h at room temperature in PBST, protected from light.

### Fluorescence imaging and analysis

Imaging of NALP5-stained oocytes was performed on an Andor Dragonfly 600 spinning disk confocal microscope (Oxford Instruments, Oxford, UK), at the Eugin Clinic’s Basic Research Laboratory, Parc Científic de Barcelona, Spain, or a Zeiss Airyscan confocal microscope (Carl Zeiss, Oberkochen, Germany) at the Babraham Institute imaging facility. All other immunofluorescence imaging was performed at the Babraham Institute imaging facility using a Zeiss LSM 780 confocal (Carl Zeiss). Images were processed using ImageJ Fiji (Schindelin *et al.*, 2012) to quantify fluorescence intensities. Fluorescence values were measured for the nucleus, cytoplasm, and background fluorescence for each oocyte image. Fluorescence plots were normalized by α or γ-tubulin fluorescence and/or background fluorescence. Details on data presentation and significance (*P*-values) can be found in the figure legends. For assessment of non-surrounded nucleolus (NSN) and SN ratio differences, the proportion of oocytes in each stage was calculated for each mouse biological replicate, and boxplots were generated using ggplot2 (version 3.4.4; Wickham, 2016). Significance was calculated using a *t*-test within the stat_compare_means function from the ggpubr package (version 0.6.0; Kassambara, 2024). Details on data presentation and significance (*P*-values) can be found in the figure legends.

### Time-lapse imaging of embryos

IVF for time-lapse imaging of developing embryos was performed at the BSU. Oocytes were collected from superovulated 4- to 10-week-old females of *Nlrp5^+/+^*, *Nlrp5^+/-^* or *Nlrp5^-/-^* genotypes. They were cleaned in M2 medium with added hyaluronidase (Sigma-Aldrich), and then IVF was performed with C57BL6/Babr sperm. Fertilized embryos were cultured in EmbryoMax KSOM medium (Merck, Darmstadt, Germany) under humidified conditions with 5% CO_2_ air composition at 37°C for 72 h in the Geri+ time-lapse incubator (Genea Biomedx, Sydney, Australia), and imaged at 5-min intervals.

### Mass spectrometry

Mass spectrometry analysis of GV oocyte samples was performed at the Babraham Institute Mass Spectrometry facility. Briefly, 3–4 bulk oocyte samples per genotype (33 GV oocytes per bulk sample) were solubilized in sodium dodecyl sulphate polyacrylamide gel electrophoresis (SDS-PAGE) loading buffer and run approximately 5 mm into SDS-PAGE gels. After Coomassie staining, gel pieces containing the entire protein-containing region from each lane were excised, and half of each band was de-stained, reduced, carbamidomethylated, and digested with trypsin (Merck, Darmstadt, Germany). 20% of the resulting peptide digests were separated using an Ultimate 3000 nanoHPLC (Thermo Fisher Scientific) on a reversed-phase column (0.075 × 500 mm, Reprosil C18AQ, 3 µm particles, Thermo Fisher Scientific) with a 120 min linear gradient from 2% to 35% acetonitrile, containing 0.1% formic acid, at a flow rate of 300 nl/min. The column was interfaced to an Orbitrap Eclipse mass spectrometer (Thermo Fisher Scientific) operating in data-independent acquisition mode (DI-AA). The mass spectrometric data were searched against the Uniprot canonical mouse proteome database (version July 2022) using DIA-NN software (version 1.8.1; Demichev *et al.*, 2020), with further processing of the results in Perseus (Rudolph and Cox, 2019).

### DNA and RNA isolation

DNA and RNA were harvested from individual oocytes using the Smart-seq2-based G&T-seq protocol (Angermueller *et al.*, 2016).

### Single-cell RNA-sequencing library preparation

cDNA libraries (product of the G&T-seq protocol) were processed into single-cell (sc)RNA-sequencing libraries using the Nextera XT DNA Library Preparation Kit (Illumina, San Diego, CA, USA. FC-131-1096) according to the manufacturer’s protocol for RNA-sequencing libraries. Library concentrations were quantified on the Bioanalyzer (Agilent Technologies, Santa Clara, CA, USA) and via the KAPA Library Quantification Kit (KAPA Biosystems, Wilmington, MA, USA). Both values were averaged, and the samples were pooled at a volume and concentration suitable for sequencing.

### scPBAT-sequencing library preparation

Single-cell methylation analysis of oocytes was performed as previously described (Castillo-Fernandez *et al.* 2020), with five rounds of first-strand synthesis. Library concentrations were quantified on the Bioanalyzer and via the KAPA Library Quantification Kit. Both values were averaged, and the samples were pooled at a volume and concentration suitable for sequencing.

### ScPBAT and scRNA sequencing

All scPBAT and scRNA-seq libraries were sequenced on an Illumina NextSeq500 High Output sequencer in 150 base pair (bp) paired-end mode, at the Babraham Institute Genomics facility.

### Proteome analysis

Using Perseus software, the result from DI-AA for *Nlrp5*^+/+^, *Nlrp5^+/^*^−^ and *Nlrp5*^−/−^ samples were plotted using principal component analysis (PCA) to determine if there was genotype-based clustering, then processed into a list of log-normalized protein abundances for each protein detected, per sample (Perseus standard pipeline, including *t*-test for comparison between genotype groups, with significance cut-offs off Benjamini–Hochberg adjusted *P*-value (false discovery rate (FDR) < 0.1 and absolute log_2_ fold change (FC) > 0.5). Differentially abundant proteins (DAPs) were plotted on volcano plots. Significance cut-offs/parameters are detailed in the figure legends. Log-normalized protein abundance counts were plotted using the Heatmap function of the ComplexHeatmap R package (Gu, 2022), version 2.14.0. First, all isoforms were plotted raw to visualize overall abundance of the isoform. Next, the per-isoform mean across samples was subtracted from each value to visualize the relative changes in abundance between samples. Where isoforms were subset, the heatmap calculation (and thus the clustering internal to the method) was performed on only the subset. Mass spectrometry datasets from two other KO studies were integrated into this analysis and compared with the *Nlrp5^+/^*^−^ and *Nlrp5*^−/−^ proteomics data (*Nlrp14* KO (Zhang *et al.*, 2023) and *Tle6* and *Padi6* KOs (Jentoft *et al.*, 2023)). These datasets were chosen for comparative analysis due to either their putative or known membership of the SCMC. The relevant datasets were downloaded from the supplemental material of their associated papers, acquired from the PRIDE protein database, or acquired directly from the authors. Enrichment analyses were performed in R (version 4.3.3) using gprofiler (Kolberg *et al.*, 2023). Further comparative analysis between protein datasets was performed in R using the pcaMethods package (ppca method) (Stacklies *et al.*, 2007).

### Sequencing data processing and quality control

Quality control (QC) and data processing were performed by the Bioinformatics team at the Babraham Institute. For scRNA-seq data processing, the raw data files were processed using the Babraham Institute Bioinformatics team’s standard nf_rnaseq pipeline (Andrews, 2023). This pipeline processes FastQ files, performs read count QC, contamination QC, quality-/adapter trimming using Trim Galore!, and splice-aware alignments to the GRCm39 mouse genome using HISAT2. Finally, it generates an aggregate QC report. For scPBAT sequencing data, the Babraham Institute nf_scBSseq pipeline was used for processing (Andrews, 2020), with the additional parameters—trim_galore_args=‘—clip_r1 9—clip_r2 9’ to account for potential mis-priming issues from the PBAT. The nf_scBSseq workflow runs an entire single-cell Bisulphite-seq (scBS-seq) processing pipeline on FastQ files. This includes QC, quality-/adapter trimming using Trim Galore!, contamination QC (post-trimming), alignments to the GRCm39 mouse genome using Bismark, deduplication, methylation extraction, and coverage file generation. Finally, it generates an aggregate MultiQC report. The workflow is usually carried out in ‘—single_end’ mode, even for paired-end libraries (Smallwood *et al.*, 2014).

### Analysis of scRNA-seq data

RNA-seq samples with fewer than 4 million reads were filtered out. The remaining samples were screened by the standard MultiQC report function of SeqMonk (Andrews, 2022) and determined to be of good quality (mitochondrial genes <2%, ribosomal rRNA <10%, 37–50% genes measured, >70% uniquely mapped reads in each sample). The oocyte samples were further subdivided into two maturity groups (NSN and SN) based on the expression of NSN and SN-specific marker genes (Supplementary File S1 (tab i) (Demond *et al.*, 2024)). Samples that could not be clearly identified as either NSN or SN stage based on these marker genes were excluded, leaving eight NSN and eight SN samples to analyse (NSN = 4 *Nlrp5*^−/−^, 4 *Nlrp5*^+/+^ (wild type; WT), SN = 2 *Nlrp5*^−/−^, 6 *Nlrp5*^+/+^ (WT)). Differentially expressed genes (DEGs) between WT and *Nlrp5* homozygous KOs were determined for each maturity group separately, using the DESeq2 package in R (Love *et al.*, 2014) (significance FDR <= 0.05; log_2_ FC >= 0.5). PCA plots were made for each maturity group separately to indicate genotype-based clustering of samples. DESeq2 results were plotted as volcano plots, using the DESeq calculated adjusted *P*-value (Benjamini–Hochberg-corrected *P*-value) and DESeq2-calculated log_2_ FC. All genes were plotted, and genes with an absolute log FC greater than 2 and an FDR of less than 10% were plotted as significant (in red, and contributing to the numeric counts at the top of the volcano plot). The 10 most significant (lowest adjusted *P*-value) genes were additionally labelled (when there were at least 10 significant hits). DEGs from both NSN and SN groups were combined (a total of 584 unique DEGs) for the comparison with other omics datasets (proteomics/methylation). Gene enrichment analyses performed in R using gprofiler.

### Analysis of scPBAT data

Processed data were analysed in SeqMonk. Processed methylation files were loaded into SeqMonk and methylation profiles for all libraries were compared to a wild-type oocyte methylome reference dataset (Castillo-Fernandez *et al.*, 2020) using the genome viewer, to estimate coverage and rule out somatic cell DNA contamination (unlike somatic cells, oocytes have a characteristic methylation pattern of hyper and hypomethylated regions, with hypermethylated regions overlapping with transcriptional units). Quality control was performed on the scPBAT samples, filtering out samples with low read count (<600,000 cytosine–guanine dinucleotides; CpGs covered) and samples with presumed somatic cell DNA contamination. Contaminated samples were inferred by three independent methods: (1) high methylation of X chromosome CpG islands (>10%), which are hypomethylated in normal, non-contaminated oocytes; (2) modified proportion of the methylated cytosines in different contexts (taken from the Bismark QC output) compared to the normal ratio of CG <40%, CHG >3%, CHH > 4% (Shirane *et al.*, 2013); (3) modified histograms of the methylation of 100-CpG tiles compared to the standard bimodal profiles, peaking at 0% and 100% of methylation, with quantification over 100 CpG windows, using methylation pipeline with minimum of five calls per probe for individual oocytes. Only oocytes with satisfactory parameters to pass all three screening criteria were retained for further analysis. No *Nlrp5^+/^*^−^ samples had sufficient coverage after MultiQC report assessment to proceed with downstream analyses. Between *Nlrp5*^+/+^ (WT) and *Nlrp5*^−/−^ samples, there was no difference in the proportion of oocytes inferred as being contaminated.

The remaining WT and *Nlrp5*^−/−^ oocyte samples were pseudo-bulked in SeqMonk (10 WT and 19 *Nlrp5*^−/−^ samples), resulting in a final CpG coverage of 28 million and 31.6 million reads for WT and *Nlrp5*^−/−^ groups, respectively. Analysis of the global DNA methylation was performed in SeqMonk, using initial quantification of probes corresponding to contiguous 100-CpG tiles across the genome, with a minimum of 30 calls per tile required for inclusion in analysis, resulting in 231 959 100-CpG tiles included. The 100-CpG tiles were subsequently filtered for analysis of different genomic features: either contained within hypo/hypermethylated domains (152 071 probes; hypomethylated domains are consecutive tiles merged with methylation of 0–25%; hypermethylated domains merged tiles of 75–100%, as detailed in Veselovska *et al.* 2015), or overlapping with hypermethylated CpG islands (>75% methylation at least in one group, WT or *Nlrp5*^−/−^; 1796 probes). Identification of differentially methylated regions (2452 hits, overlapping with 1402 genes) amongst the 100-CpG tiles contained within hypo/hypermethylated domains was carried out using a chi-square test with significance cut-offs of the adjusted *P*-value <0.05 and % methylation difference of >25%. The differential methylation of germline differentially methylated regions (gDMRs) and their heatmap were analysed across whole gDMRs (with quantification of minimum of 20 calls per gDMR). The gDMRs were identified with chi-square test with the above cut-offs on the significance. gDMRs were only considered significantly differentially methylated if also highly covered (>100 CpG calls/1000 bp).

### Multi-omic analysis

To determine whether there was a significant overlap between scRNA-seq and proteomics hits (DEGs and DAPs, respectively), hypergeometric tests were performed (indicating *P*-value for overlaps) in R. Transcriptomic and proteomic log_2_ FCs were plotted against one another.

## Results

### Whole cytoplasm localization of NALP5 in germinal vesicle oocytes

**Figure 1.**
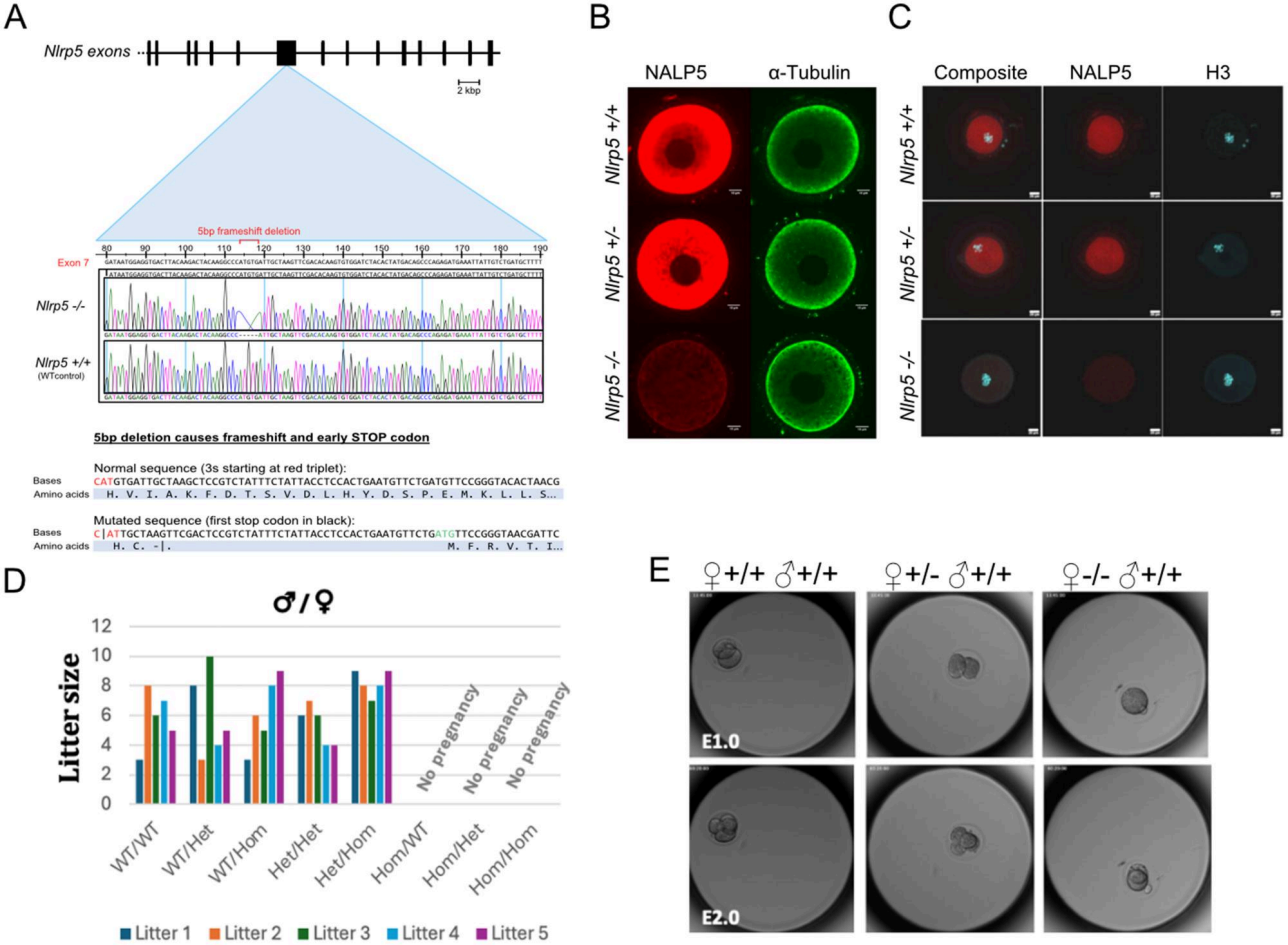
***Nlrp5* knockout mouse design and characterization**. (**A**) Schematic of target sequence (base and amino acid mutation) for CRISPR/Cas9-mediated knockout generation, (**B**) Representative images showing immunofluorescence of NALP5 protein (red) in germinal vesicle (GV) *Nlrp5*^+/+^, *Nlrp5*^*+/*^^−^, and *Nlrp5*^−/−^ oocytes. α-tubulin staining control (green). Scale bar, 10 µm. n = 6 *Nlrp5*^−/−^, 6 *Nlrp5^+/^*^−^, 4 *Nlrp5*^+/+^. (**C**) Representative images showing immunofluorescence of NALP5 protein (red) in Triton X-100 extracted GV oocytes; Histone H3 staining control (blue). Scale bar, 20 µm. n = 8 *Nlrp5*^−/−^, 6 *Nlrp5^+/^*^−^, 2 *Nlrp5*^+/+^. (**D**) Clustered column chart showing the number of pups per litter for mating pairs of each genotype combination. No pregnancies in matings with *Nlrp5*^−/−^ females. WT, *Nlrp5*^+/+^; Het, *Nlrp5^+/^*^−^; Hom, *Nlrp5*^−/−^. (**E**) Representative time-lapse images of wild-type and *Nlrp5* mutant embryos at embryonic day (E)1.0 and E2.0, demonstrating two-cell arrest of maternal *Nlrp5* knockout-derived embryo (n = 16 ♀ *Nlrp5*^+/+^, 11 ♀*Nlrp5^+/^*^−^, 16 ♀ *Nlrp5*^−/−^).

*Nlrp5* encodes the NALP5 protein, which when absent or severely depleted results in the SCMC losing its structural integrity (Lu *et al.*, 2017). Severe depletion of CPLs (Kim *et al.*, 2010), an increase in disordered mitochondrial localization and activity (Fernandes *et al.*, 2012), and altered endoplasmic reticulum distribution and calcium homeostasis (Kim *et al.*, 2014) in murine oocytes have also been reported. *Nlrp5-*deficient oocytes from previous models lead to embryonic arrest at the early cleavage stages when fertilized by WT sperm (Tong *et al.*, 2000; Yurttas *et al.*, 2008). To interrogate the consequences of SCMC disruption on oocyte and pre-implantation embryo development, we generated a new KO mouse model with an inactivating deletion in the core SCMC gene *Nlrp5.* We employed CRISPR/Cas9 technology to create an *Nlrp5* KO by inducing a double-stranded break within the *Nlrp5* gene, which led to a 5 bp deletion, on a C57BL6/Babr genetic background. Our deletion, hereby referred to as *Nlrp5*^−/−^, produced a premature stop codon, causing the mRNA to be targeted for nonsense-mediated decay (Fig. 1A). Ablation of the NALP5 protein was tested in *Nlrp5^+/^*^−^ and *Nlrp5*^−/−^ GV-stage oocytes via confocal immunofluorescence imaging. Immunofluorescence of fixed GV oocytes demonstrated that NALP5 protein is ablated in *Nlrp5*^−/−^ oocytes, and in *Nlrp5^+/^*^−^ oocytes the concentrated subcortical signal for NALP5 observed in *Nlrp5*^+/+^ oocytes is diminished and becomes more evenly distributed throughout the cytoplasm (Fig. 1B). Immunofluorescence analysis of Triton X-100 extracted GV oocytes showed a uniform signal throughout the cytoplasm for NALP5 in both *Nlrp5*^+/+^ and *Nlrp5^+/^*^−^ oocytes, and undetectable signal in *Nlrp5*^−/−^ oocytes (Fig. 1C). Although the term SCMC was coined because of the apparent immunofluorescence localization of its constituent proteins to the oocyte subcortex, this observation, coupled with recent colocalization studies (Kim *et al.*, 2010; Jentoft *et al.*, 2023), suggests that the observed subcortical localization of these proteins is likely an artefact of oocyte permeabilization and staining protocols leading to oversaturation with secondary antibodies at the subcortex (Morency *et al.*, 2011).

### Impaired nuclear maturation and developmental competence of *Nlrp5*^−/−^ oocytes is accompanied by differences between *Nlrp5*^−/−^ and *Nlrp5*^+/+^ oocyte transcriptomes

*Nlrp5* ^−/−^ and *Nlrp5^+/^*^−^ mice were phenotypically indistinguishable from *Nlrp5*^+/+^ mice in soma, though *Nlrp5*^−/−^ females were infertile, as previously reported (Tong *et al.*, 2000). Inter-genotype crosses set up to determine if mutants had differences in fertility or litter sizes showed that *Nlrp5*^−/−^ males and *Nlrp5^+/^*^−^ mice of either sex had normal fertility, and litter sizes were not significantly different from wild-type pairings (Fig. 1D). IVF was performed on oocytes collected from 4- to 10-week-old *Nlrp5*^−/−^, *Nlrp5^+/^*^−^, and *Nlrp5*^+/+^ mice, fertilized with wild-type C57BL6/Babr sperm. Fertilized zygotes were cultured in a time-lapse imaging incubator for 72 h, showing that our *Nlrp5* KO recapitulated the two-cell embryonic arrest phenotype reported in previous *Nlrp5* mutants (Tong *et al.*, 2000; Fernandes *et al.*, 2012). Fertilized *Nlrp5*^−/−^ oocytes did not progress past the two-cell stage of embryogenesis (Fig. 1E), with most arresting or becoming visibly degenerated prior to the first cell division. In several cases, embryos that did achieve the two-cell stage underwent reverse cleavage before degenerating. *Nlrp5^+/^*^−^ oocyte-derived embryos developed as normal.

**Figure 2.**
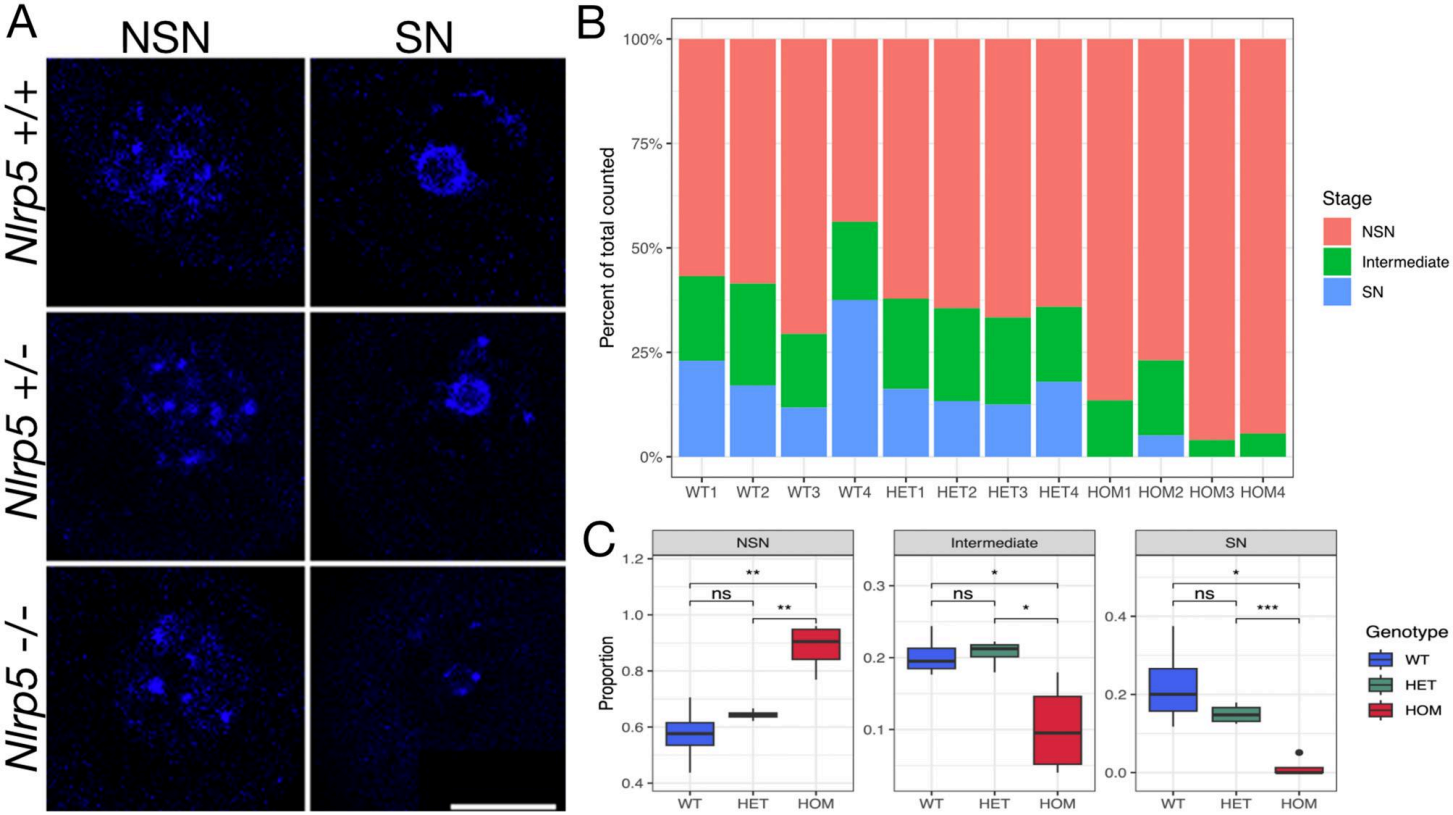
**Analysis of nuclear maturation in *Nlrp5*** ^−/−^  **oocytes**. (**A**) Representative images of Hoechst-stained non-surrounded nucleolus (NSN) and surrounded nucleolus (SN) *Nlrp5*^−/−^, *Nlrp5*^*+/*^^−^, and *Nlrp5*^+/+^ oocytes. Scale bar, 20 µm. (**B**) Proportions of germinal vesicle (GV) oocytes of each stage per biological replicate (4 mice per genotype, 17–89 GV oocytes classified per replicate). (**C**) Proportion of GV oocytes of each stage per genotype group. Boxes represent the interquartile range (IQR; 25th–75th percentiles) with the horizontal line indicating the median. Whiskers extend to the most extreme data points within 1.5× IQR from the box; individual points beyond these limits are plotted as outliers. *t*-test significance *P < 0.05, **P < 0.01, ***P < 0.001, ns = not significant. WT, *Nlrp5^+/+^*; HOM, *Nlrp5*^−/−^; HET, *Nlrp5^+/^*^−^.

During the GV stage of oocyte maturation, chromatin undergoes a conformational change, moving from the less mature NSN stage to the more mature SN stage, at which point the oocyte becomes largely transcriptionally silent (Zuccotti *et al.*, 1995; Bogolyubova *et al.*, 2023). Although oocytes may bypass the NSN–SN transition during maturation, oocytes that have not successfully achieved the SN checkpoint typically lead to cleavage-stage embryonic arrest when fertilized (Monti *et al.*, 2013). Upon fluorescence imaging of Hoechst-stained GV oocytes, we observed that very few *Nlrp5*^−/−^ oocytes achieved the SN stage of oocyte nuclear maturation, and those that did had a qualitatively different SN conformation than *Nlrp5^+/^*^−^ and *Nlrp5*^+/+^ oocytes (Fig. 2A, B, and C). This nuclear maturation impairment was detectable in oocytes from *Nlrp5*^−/−^ females as young as 3 weeks old. There were no significant differences between *Nlrp5^+/^*^−^ and *Nlrp5*^+/+^ oocytes (Fig. 2B and C).

**Figure 3.**
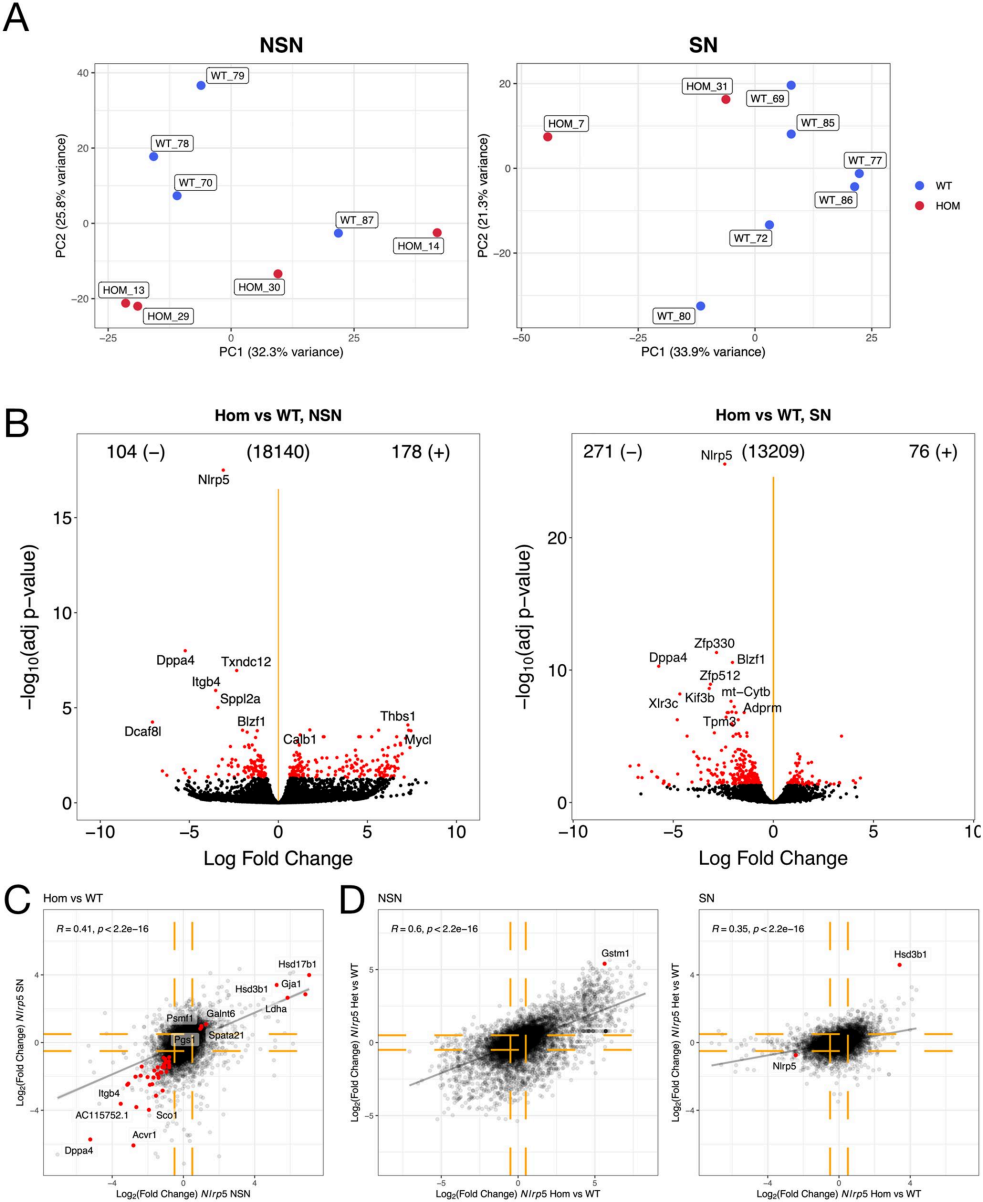
**Comparison of *Nlrp5*** ^−/−^  **and *Nlrp5***^+/+^  **oocyte transcriptomes, accounting for non-surrounded nucleolus (NSN)/surrounded nucleolus (SN) staging**. (**A**) Principal component analysis of *Nlrp5*^+/+^ (WT) and *Nlrp5*^−/−^ (HOM) samples (all expressed genes), after assignment to NSN (left) and SN (right) stages. (**B**) Volcano plots showing *Nlrp5*^−/−^ differentially expressed genes (DEGs) at NSN (left) and SN stage (right), respectively. Significant DEGs highlighted in red (significant where log_2_ fold change is <−0.5 or >0.5, and Benjamini–Hochberg (BH)-adjusted *P*-value is <0.05), (**C**) Scatter plot showing correlation of log_2_ fold change gene expression for all genes detected in *Nlrp5*^−/−^ samples, comparing expression at NSN and SN stages. DEGs significant in both stages are highlighted in red (45 shared DEGs). FDR <= 0.05, log_2_ fold change >= 0.5 in either or both NSN and SN groups, *R* (correlation coefficient) = 0.41. Yellow dashed lines show absolute log_2_ fold change > 0.5. (**D**) Scatter plots showing positive correlation of log_2_ fold change gene expression in *Nlrp5*^−/−^ and *Nlrp5^+/^*^−^ samples, compared with *Nlrp5*^+/+^ samples. NSN and SN stages are plotted separately. *R* (correlation coefficient) = 0.6 and 0.35, respectively. DEGs significant in both *Nlrp5^+/^*^−^ and *Nlrp5*^−/−^ cohorts are highlighted in red. Yellow dashed lines show absolute log_2_ fold change > 0.5.

To detect differences in gene expression that occur between comparably staged GV oocytes, we collected GV oocytes from 3-week-old females of each genotype and performed scRNA sequencing. Because of the observed impairment of nuclear maturation in *Nlrp5*^−/−^ oocytes, NSN/SN staging was inferred bioinformatically using a list of NSN–SN stage-specific DEGs (Demond *et al.*, 2024) (Supplementary Fig. S1, Supplementary File S1 (tab i)). After filtering samples for minimum read count, between 2 and 6 samples per stage and genotype remained for analysis (Supplementary Fig. S2A). There was no difference in the mean number of genes detected in *Nlrp5*^−/−^ or *Nlrp5^+/^*^−^ oocytes at either stage (Supplementary Fig. S2B). Global transcriptomic analyses demonstrated that *Nlrp5*^−/−^ oocytes could be separated from *Nlrp5*^+/+^ oocytes of the equivalent NSN/SN stage (Fig. 3A), although when staging is not considered, this distinction is obscured. DEGs detected between *Nlrp5*^−/−^ and *Nlrp5*^+/+^ oocytes (log_2_ FC <−0.5 or >0.5, Benjamini–Hochberg-adjusted *P*-value <0.05) accounted for <3% of expressed genes: 282/18140 (1.55%) in NSN and 347/13209 (2.63%) in SN oocytes (Fig. 3B, Supplementary File S1 (tab ii)).

DEGs were distributed across the whole range of transcript abundances (Supplementary Fig. S3). Taken together, this suggests that disruption of the SCMC in the absence of NALP5 is unlikely to lead to major effects on mRNA stability in GV oocytes, though some transcript specific effects on stability could explain a higher number of DEGs being downregulated in the more mature, transcriptionally inert, SN-stage oocytes than NSN oocytes (Fig. 3B). Although only 45 DEGs were called in common between NSN and SN stages, differential expression of DEGs at either stage was mostly correlated between stages (Pearson correlation, *R* = 0.41, *P* < 2.2e^−16^; Fig. 3C).

Far fewer DEGs were detected between the *Nlrp5^+/^*^−^ and *Nlrp5*^+/+^ oocytes using the cut-offs above (Supplementary File S1 (tab iii)), although FC in transcript abundances in the heterozygotes was positively correlated with FC in the homozygotes (Pearson correlation, *R* = 0.6, *P* < 2.2e^−16^ in NSN, *R* = 0.35, *P* < 2.2e^−16^ in SN; Fig. 3D, Supplementary Fig. S4). This indicates that a certain level of transcriptome misregulation is detectable in the *Nlrp5^+/^*^−^ oocytes, but given that *Nlrp5^+/^*^−^ oocytes can be successfully fertilized to produce viable embryos, the transcriptome misregulation detected does not reflect impaired developmental competence of heterozygote oocytes.

Gene enrichment analysis highlighted general terms such as ‘cellular response to stimulus’, ‘steroid biosynthesis’, and ‘cellular anatomical entity’ in upregulated NSN-stage *Nlrp5*^−/−^ DEGs (Supplementary Fig. S5A). The ‘Hsp110–Hsc70–Hsp25’ heat shock protein complex was enriched in upregulated DEGs at the SN stage, in addition to ‘steroid biosynthesis’ (Supplementary Fig. S5B). Terms related to DNA binding and transcriptional regulation were enriched in downregulated DEGs at the SN stage (Supplementary Fig. S5C). Apart from strong down-regulation of *Nlrp5* (Fig. 3B), which might indicate nonsense-mediated decay of the mutant transcript, there was no evidence for altered mRNA abundance of components of the SCMC. And, other than *Dppa4*, no transcripts coding for known epigenetic modifiers were significantly differentially abundant (Supplementary File S1 (tab ii)).

### Proteomic analysis shows misregulation of a subset of SCMC proteins in *Nlrp5*^−/−^ GV oocytes

Mass spectrometry analysis was performed on *Nlrp5*^−/−^, *Nlrp5^+/^^−^*, and *Nlrp5^+/+^* GV oocytes to determine if causes of the developmental defect could be detected in the proteome. GV oocytes were collected from 3-week-old mice in bulk samples (33 oocytes per sample), and the gel-liquid chromatography–tandem mass spectrometry analysis used data-independent tandem mass spectrometry (MS/MS) mode. The data were searched against the Uniprot canonical mouse proteome database using DIA-NN software. Over 5228 proteins were identified per sample (Supplementary Fig. S6, Supplementary File S2 (tab i)). *Nlrp5*^−/−^ bulk oocyte samples showed a log_2_ FC for NALP5 of −8.47 compared with *Nlrp5*^+/+^ oocytes, which corresponds to 0.3% of the *Nlrp5*^+/+^ level. *Nlrp5^+/^*^−^ samples had a far lower but still significant log_2_ FC of −0.55, corresponding to 68% of the *Nlrp5*^+/+^ NALP5 level (Table 1). *Nlrp5*^−/−^ samples separate out from *Nlrp5*^+/+^ samples based on global protein abundances, with *Nlrp5^+/^*^−^ samples overlapping with both groups (Supplementary Fig. S6, Supplementary File S2 (tabs i and ii)). To test whether the effects seen in the *Nlrp5*^−/−^ proteomics data could be attributed to a staging skew, the data were compared to existing wild-type SN/NSN proteomics data (Monti *et al.*, 2013); however, no significant difference in abundance in proteins that were known to be differentially abundant in NSN compared to SN was found (Supplementary Fig. S7).

**Table 1.** Raw abundance values for subcortical maternal complex (SCMC) and putative SCMC proteins in the *Nlrp5* proteomics dataset.

SCMC protein	*Nlrp5^+/+^*				***Nlrp5*** ^*+/*^^−^			***Nlrp5*** ^−/−^			
	WT1*	WT2	WT3	WT4	Het1	Het2	Het3	Hom1	Hom2	Hom3	Hom4
**NALP5**	29249613	22990500	21701309	34329594	20580396	17248192	16886991	53745	48954	81185	148701
**KHDC3**	11967394	12713007	14098895	17847493	11147194	10542905	10571504	2788520	2913010	2819019	3640121
**NAL14**	16254890	12680208	14625793	16085598	16003190	14473409	14968203	12309301	13283708	13467793	13275492
**NAL4A**	754523	699619	687737	711488	611843	748684	663539	661840	623275	561566	523258
**NAL4B**	610404	650773	594686	822934	440422	559545	366959	102046	106833	104870	117434
**NAL4C**	59274	49927	51552	44729	54071	57460	50931	54313	58425	55263	51772
**NAL4E**	352690	540195	327480	468682	385182	482290	462373	230875	269931	295463	254706
**NAL4F**	12823795	11083006	11202093	11663105	10163901	9370426	9485609	4863791	5278447	4791200	4382168
**NLR9A**	259508	270431	263361	353803	279618	289361	279132	151600	176139	191157	195920
**NLR9B**	1870511	1754859	1775700	2211160	1795169	1794531	1793579	1311269	1523149	1630670	1640971
**NLR9C**	24646	26505	27227	31581	25771	27969	30852	20928	19472	23700	23276
**OOEP**	18745907	19388711	17366810	33067496	14524897	12651607	12976194	1400471	1534961	1522400	3306391
**PADI6**	7054146	4412748	6474884	7464388	8012424	6238770	7107938	6630764	6899897	8170453	7919411
**Q4PLS0**	3047962	2794192	2727011	3655259	3081808	3236941	3002770	2132909	2539420	2663170	2545841
**TLE6**	26003597	19599088	23813490	28516604	21401997	19820604	18771899	3313901	3356508	3616430	3896881
**ZBED3**	1869001	1513690	1581990	2549981	1212480	1078780	1403240	52474	56548	65468	103955

* WT1, WT2, Het1, Het2, Hom1, Hom2, etc., denote sample names. WT, wild type or *Nlrp5*^+/+^; Het, *Nlrp*5^*+/*^^−^; Hom, *Nlrp5*^−/−^.

**Figure 4.**
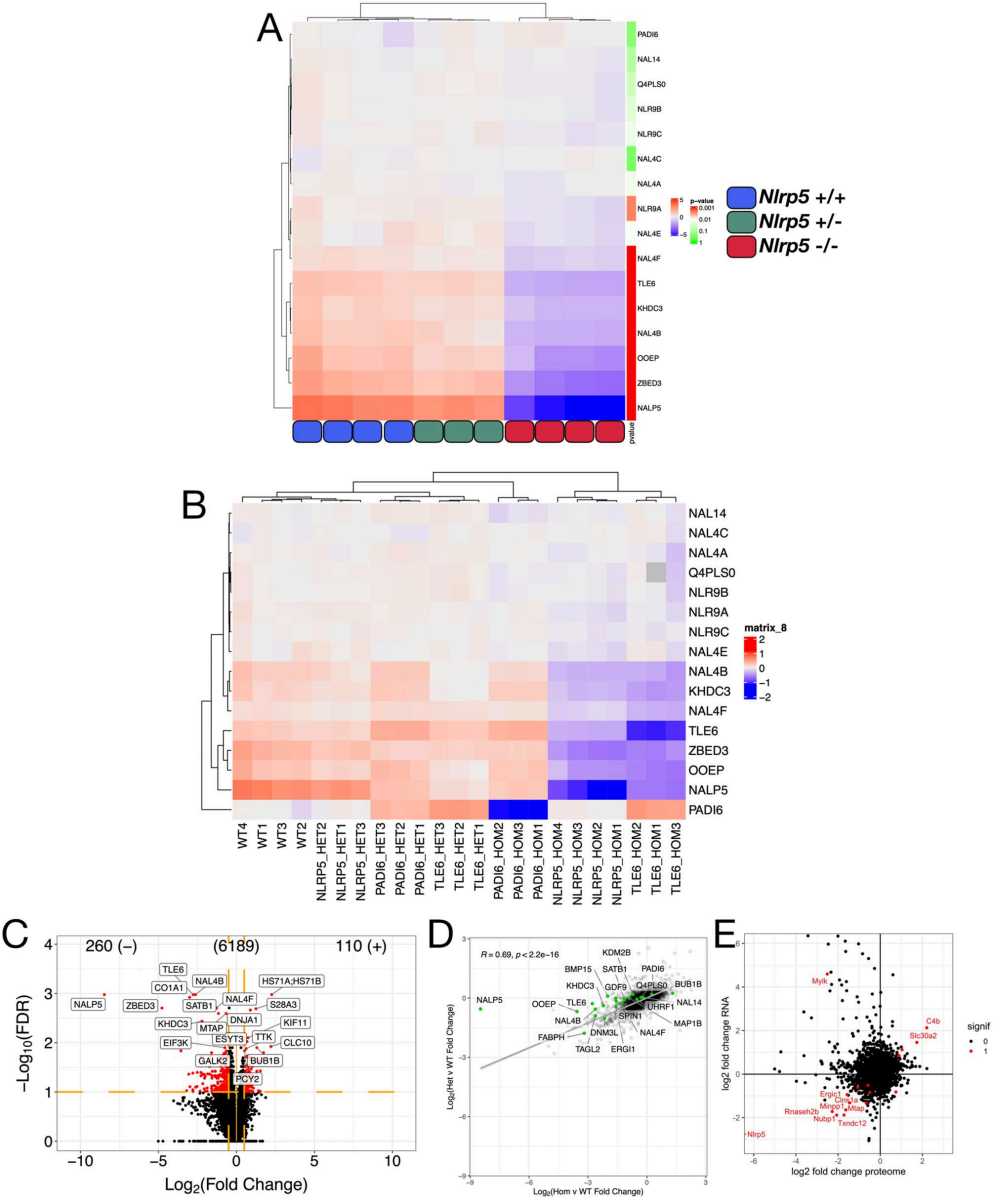
**Comparative analysis of *Nlrp5-*, *Tle6-*, and *Padi6*-null germinal vesicle (GV) oocyte proteomes**. (**A**) Hierarchically clustered heatmap showing standardized log_2_ abundance (standardized by mean across samples for a given protein, but not by variance; colour is red–white–blue scale, with red indicating above-average abundance, white centred on 0, and blue indicating below-average abundance) of protein abundances between *Nlrp5*^−/−^ (red), *Nlrp5^+/^*^−^ (green), and *Nlrp5*^+/+^ (blue) samples for known and putative subcortical maternal complex (SCMC) proteins, annotated with the *P*-value of the given protein in the *Nlrp5*^+/+^ vs *Nlrp5*^−/−^ differential abundance test (*t*-test; colour is green–white–red on a log10 scale, with white centred at *P* = 0.01). (**B**) Hierarchically clustered heatmap showing standardized log_2_ abundance (subtracting the mean and dividing by the variance across all proteins for each dataset, and further normalized by subtracting the dataset-specific row means for each protein; colour is red–white–blue scale, with red indicating above-average abundance, white centred on 0, and blue indicating below-average abundance) of protein abundances across the NALP5, TLE6, and PADI6 datasets for known and putative SCMC proteins. (**C**) Volcano plot of all differentially abundant proteins between *Nlrp5*^−/−^ and *Nlrp5*^+/+^ GV oocytes. Significant proteins highlighted in red, top 20 labelled (false discovery rate (FDR) < 0.1, log_2_ fold change of <−0.5 or > 0.5 [protein abundance altered by 30% or more]). (**D**) Scatterplot showing correlation of global protein abundances between the *Nlrp5*^−/−^ (Hom) vs *Nlrp5*^+/+^ (WT) and *Nlrp5^+/^*^−^ (Het) vs *Nlrp5*^+/+^ (WT) comparisons (log_2_ fold changes). Proteins of interest highlighted in green. (**E**) Scatterplot of global protein vs transcriptomic (RNA) changes (log_2_ fold changes). Significant hits in both datasets are highlighted in red.

When *Nlrp5*^−/−^, *Nlrp5^+/^*^−^, and *Nlrp5*^+/+^ samples were clustered based on abundances of SCMC proteins, *Nlrp5*^−/−^ samples separate out strikingly from *Nlrp5^+/^*^−^ and *Nlrp5*^+/+^ samples (Fig. 4A), although similar trends in SCMC protein abundances apply in the *Nlrp5^+/^*^−^ samples, but to a lesser degree (Supplementary File S2 (tabs i and ii)). Significance cut-offs of log_2_ FC <−0.5 or >0.5 and FDR < 0.1 were used to determine the significance of protein abundance differences between *Nlrp5*^−/−^ and *Nlrp5*^+/+^ oocyte samples. The −8.47 log_2_-fold reduction of NALP5 in *Nlrp5*^−/−^ samples is associated with a significant reduction in the abundances of seven known or putative components of the SCMC: TLE6, OOEP, ZBED3, KHDC3, NLRP4f, NLRP4b, and NLRP9a (Fig. 4A, Table 1). Of these, NLRP4b and NLRP9a have not previously been demonstrated to be part of the SCMC. The strong reduction (>85%) in TLE6 and OOEP, two proteins considered necessary for the complex to form (Bebbere *et al.*, 2021), at the protein level but not at the transcriptome level, demonstrates that the integrity of the complex is strongly affected by the ablation of NALP5. Notably, PADI6, NLRP2 (Q4PLS0), NLRP14, and five other NLRPs detected were not significantly changed in abundance in the *Nlrp5*^−/−^ samples (Fig. 4A).

Comparison of our *Nlrp5* proteomic data with published *Tle6-* and *Padi6*-null datasets (Jentoft *et al.*, 2023) shows greater similarities between the *Nlrp5*^−/−^ and *Tle6*^−/−^ proteomes than comparisons of either with the *Padi6*^−/−^ proteome, when comparing log-normalized abundances of known and putative SCMC proteins (Fig. 4B). This suggests two functionally separate (or at least partially redundant) SCMC protein subgroups. The often subtle but nonetheless measurable change in abundance of the array of NLRP4 and NLRP9 isoforms across studied SCMC mutant proteomes indicates that these proteins are SCMC-related, although they do not fit neatly into either of the two clear classes. Despite operating as separate functional subgroups to influence abundance of other SCMC-related proteins and other maternal proteins, both the NALP5–TLE6 category of SCMC proteins and the PADI6 category are thought to be necessary for the integrity of CPLs in the oocyte cytoplasm (Yurttas *et al.*, 2008; Kim *et al.*, 2010; Kan *et al.*, 2011; Qin *et al.*, 2019), and null mutations in any of these three genes have similar two-cell arrest phenotypes (Tong *et al.*, 2000; Yurttas *et al.*, 2008; Alazami *et al.*, 2015).

### Broad range of maternal proteins altered in abundance in *Nlrp5*-null oocytes

In total, 370 DAPs were identified in the *Nlrp5*-null data between *Nlrp5*^−/−^ and *Nlrp5*^+/+^ samples (Fig. 4C). However, when the same significance cut-offs were used (<0.1 FDR, log_2_ FC <= −0.5 or >= 0.5) in the *Tle6*- and *Padi6*-null datasets, only 10 DAPs were identified in the *Tle6*-null data, and 128 in the *Padi6*-null data. Further comparative analysis of the *Nlrp5*-null, *Tle6*- and *Padi6*-null proteomes was performed using these three lists of DAPs. A published list of DAPs from an *Nlrp14*-null oocyte dataset (Zhang *et al.*, 2023) was also used for comparison, where overlapping proteins were detected.

Of the DAPs between *Nlrp5*^−/−^ and *Nlrp5*^+/+^ samples, 260 were significantly reduced in abundance in *Nlrp5*^−/−^ samples and 110 were significantly increased (Fig. 4C). Comparison of *Nlrp5^+/^*^−^ and *Nlrp5*^+/+^ samples yielded no significant DAPs using the same cut-offs, however, correlation analysis performed on the log_2_ FC of all detected proteins concluded that, while not reaching the threshold for significance and with a smaller magnitude, the log_2_ FCs were positively correlated between the homozygous and heterozygous *Nlrp5* mutant oocytes (Pearson correlation, *R* = 0.69, *P* < 2.2e^−16^; Fig. 4D). Importantly, although *Nlrp5*^−/−^ and *Nlrp5*^+/+^ samples can be clearly separated based on both the transcriptome and proteome, with very few exceptions, no correlation exists between transcript and protein abundance changes (Fig. 4E). Enrichment analysis for DAPs in the *Nlrp5*^−/−^ vs *Nlrp5*^+/+^ comparison resulted in very few hits. Upregulated DAPs were enriched for ‘regulation of protein modification process’, and downregulated DAPs were enriched for ‘Khdc3–Nlrp5–Ooep–Tle6 complex’ (Supplementary Fig. S8). As very few enrichment terms emerged from the enrichment analysis of the *Nlrp5*^−/−^ DAPs, published literature describing proteins and biological processes altered in SCMC mutant oocytes, coupled with hits from comparative analyses with other SCMC mutant datasets (Jentoft *et al.*, 2023; Zhang *et al.*, 2023), were used as guidelines to investigate changes in certain protein groups within the *Nlrp5*-null dataset.

DAPs in the *Nlrp5*^−/−^ oocytes included a broad range of proteins important for a diversity of biological processes necessary for oocyte and embryo development (Supplementary File S2 (tabs iii and iv)). SCMC components have been linked to maintenance of euploidy during cleavage-stage mouse embryogenesis via regulation of microtubule-related proteins such as γ-tubulin. Significant reductions in abundances of tubulins and tubulin-associated proteins, and a reduction in acetylation of tubulins, have been described in *Padi6-* and *Nlrp14*-null oocytes (Kan *et al.*, 2011; Jentoft *et al.*, 2023; Zhang *et al.*, 2023). Three cytoskeleton-associated proteins were significantly reduced in *Nlrp5*^−/−^ samples: TBAL3, which was reduced by ∼30%; TAGL2, reduced by 80%, and MAP1B, reduced by over 40%. The only overlapping protein between the datasets was MAP1B, which was altered in both *Nlrp5-*null and *Padi6*-null oocytes. It was also listed as significantly reduced in *Nlrp14*-nulls (Zhang *et al.*, 2023). Its abundance was unchanged in *Tle6*-nulls. Despite minimal overlap in effect on abundances of specific cytoskeleton-related proteins between *Nlrp5*, *Tle6*, and *Padi6*-null datasets, there is likely to be some overlapping function in cytoskeleton architecture, particularly via microtubule organization.

Two proteins with important roles in folliculogenesis, GDF9 and BMP15, were strikingly reduced in *Nlrp5*^−/−^ oocytes (65% and 75%, respectively). GDF9 and BMP15 are thought to cooperate synergistically to influence granulosa cell proliferation and ovulation rate (Yan *et al.*, 2001), so an effect on one may influence the abundance of the other. *Gdf9*-null mice undergo arrest of folliculogenesis at the primary stage (Dong *et al.*, 1996). Interestingly, despite the strong reduction in GDF9 abundance in *Nlrp5*^−/−^ oocytes, no apparent difference in ovarian or follicular morphology, or ovulation rate in response to stimulation with exogenous gonadotrophins is observed in *Nlrp5*^−/−^ mice (Tong *et al.*, 2000). *Bmp15-*null mice do not show evidence of reduced folliculogenesis, but are subfertile due to defective ovulation and reduced viability of embryos (Persani *et al.*, 2014). Tightly controlled *Gdf9*:*Bmp15* mRNA ratio and post-translational modifications of their protein products are considered important for controlling ovulation rate in a species-specific manner (Crawford and McNatty, 2012). Although infertile, *Nlrp5*-null female mice have normal ovulation (Tong *et al.*, 2000). Despite these effects in *Nlrp5*^−/−^ oocytes, none of the other SCMC KO datasets (*Tle6-*, *Padi6*-, *Nlrp14*-nulls) showed any significant change in GDF9 or BMP15 abundance.

Several factors involved in meiotic control and chromatin organization were altered in *Nlrp5*^−/−^ oocytes, including SPIN1, SATB1, and BUB1B. Of these, SPIN1 and SATB1 were downregulated, and BUB1B was strongly upregulated. SPIN1 is a chromatin reader (Wang *et al.*, 2011) and regulator of oocyte meiotic resumption, which may act by regulating maternal transcripts (Chew *et al.*, 2013). It was reduced by over 65% in *Nlrp5*^−/−^ samples, and by over 40% in *Tle6*^−/−^ samples, but was unaltered in *Padi6*^−/−^ samples. It was not listed as significantly altered in *Nlrp14*-null oocytes. SATB1 and BUB1B were not significantly changed in *Tle6*- or *Padi6*-null datasets, and were not listed in the *Nlrp14*-null DAP list.

Other significantly altered proteins in *Nlrp5*-null oocytes included FABP3, which binds free long-chain fatty acids (LCFAs) and transports them for cell metabolism, thereby protecting against lipid toxicity (Lee *et al.*, 2020), and ERGIC1, which is involved in protein cycling and is thought to play a role in protein transport between the endoplasmic reticulum and Golgi (Breuza *et al.*, 2004). While most DAPs in the *Nlrp5*^−/−^ oocytes were downregulated, notable exceptions were heat shock chaperone proteins HS90A, HS105, HS71A, and HS71B (65–380% increase), and apoptosis-related factors, such as TP63. The latter two groups may reflect impairments in cellular regulation and reduced oocyte developmental competence. However, none of the same heat shock family proteins were differentially abundant in *Tle6-* or *Padi6-*null samples. These differences may indicate cellular and developmental defects unique to *Nlrp5*^−/−^ oocytes.

### Epigenetic modifier proteins amongst those significantly altered in abundance in *Nlrp5*-null oocytes

**Figure 5.**
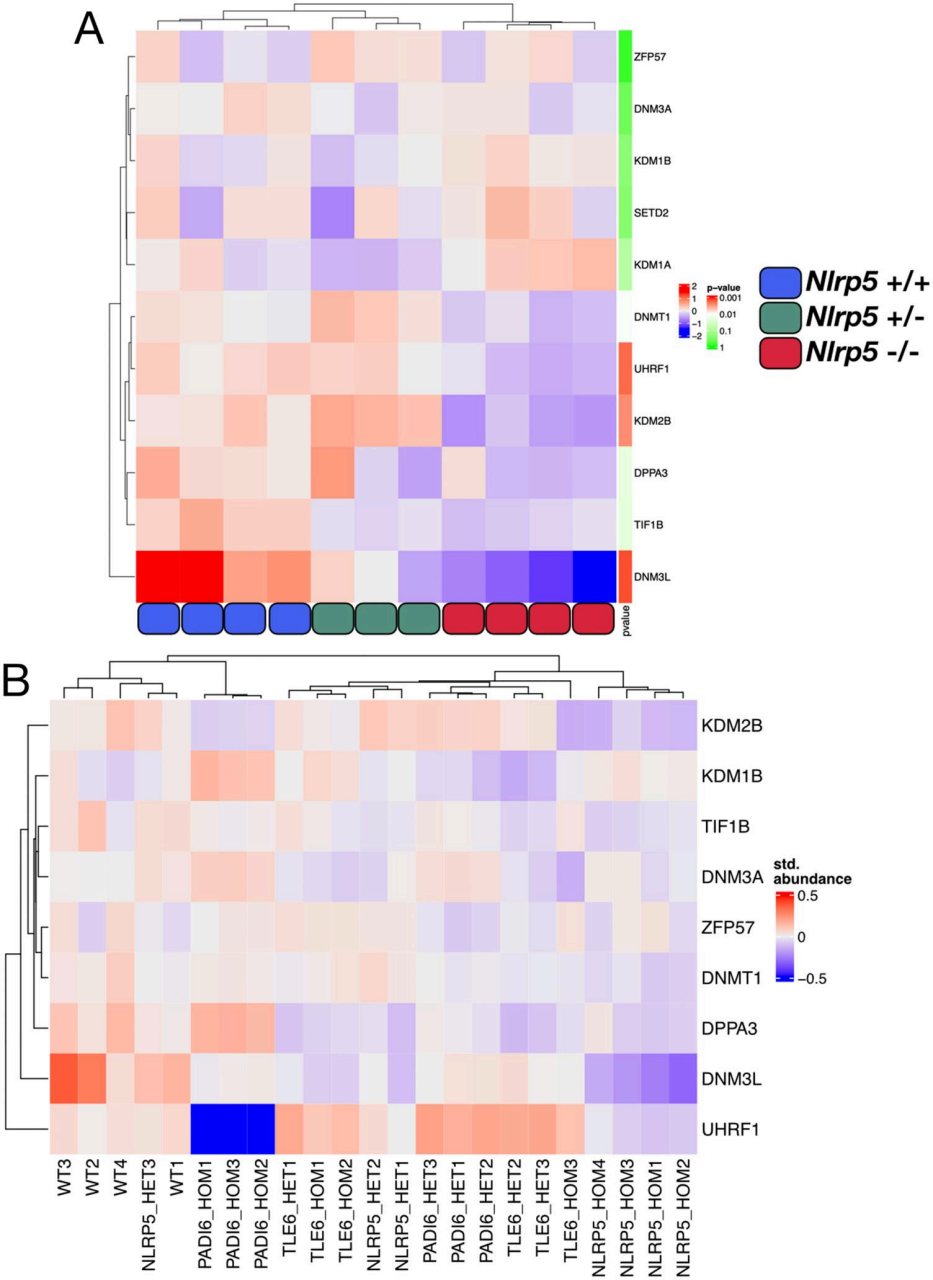
**Altered abundances of known epigenetic modifier proteins detected in *Nlrp5* mass spectrometry data**. (**A**) Hierarchically clustered heatmap showing standardized log_2_ abundances (standardized by mean across samples for a given protein, but not by variance; colour is red–white–blue scale, with red indicating above-average abundance, white centred on 0, and blue indicating below-average abundance) of protein abundances between *Nlrp5*^−/−^ (red), *Nlrp5^+/^*^−^ (green) and *Nlrp5*^+/+^ (blue) samples for known epigenetic modifier proteins, annotated with the *P*-value of the given protein in the *Nlrp5*^+/+^ vs *Nlrp5*^−/−^ differential abundance test (*t*-test; colour is green–white–red on a log10 scale, with white centred at *P* = 0.01). (**B**) Hierarchically clustered heatmap showing standardized log_2_ abundance (subtracting the mean and dividing by the variance across all proteins for each dataset, and further normalized by subtracting the dataset-specific row means for each protein; colour is red–white–blue scale, with red indicating above-average abundance, white centred on 0, and blue indicating below-average abundance) of known epigenetic modifier proteins across the NALP5, TLE6, and PADI6 datasets.

When samples were plotted based on raw abundances of 11 epigenetic modifier proteins with known roles in *de novo* methylation and methylation maintenance, *Nlrp5*^−/−^ samples cluster separately from *Nlrp5^+/^*^−^ and *Nlrp5*^+/+^ samples (Fig. 5A). While most of these proteins were not significantly altered in abundance in *Nlrp5*^−/−^ samples, three were: DNMT3L (>75% reduction compared with *Nlrp5*^+/+^), UHRF1 (30% reduction) and KDM2B (>40% reduction) (Fig. 5A, Table 2, Supplementary File S2 (tab iii)). These reductions occurred without parallel changes in transcript level (Supplementary File S1 (tab ii)). DNMT1 also appeared reduced in *Nlrp5*^−/−^ samples, but did not meet the cut-off for significance. These key proteins also show some changes in other datasets. Thus, DNMT3L, an important cofactor for DNMT3A for *de novo* DNA methylation activity in the mouse, is reduced by 30% in *Padi6*^−/−^ oocytes (Jentoft *et al.*, 2023), but not in *Tle6*^−/−^ oocytes (Supplementary File S2 (tabs v and vi)). UHRF1 is a factor important for maintenance of DNA methylation via the localization of DNMT1 to hemi-methylated sites of the DNA during the S phase of DNA replication (Sharif *et al.*, 2007). Its abundance was not significantly affected in *Tle6*-null GV oocytes, but it is strongly reduced in *Padi6-*null (Fig. 5B, Supplementary File S2 (tabs v and vi)) and *Nlrp14*-null oocytes (Zhang *et al.*, 2023). KDM2B, a histone H3 demethylase known to target H3K36me2, was significantly reduced in *Nlrp5*-null as well as in *Padi6*-null oocytes, but unchanged in *Tle6-*nulls (Fig. 5A and B).

**Table 2.** Raw abundance values for selected epigenetic modifier proteins in the *Nlrp5* proteomics dataset.

Epigenetic modifiers	*Nlrp5^+/+^*				***Nlrp5*** ^*+/*^^−^			***Nlrp5*** ^−/−^			
	WT1*	WT2	WT3	WT4	Het1	Het2	Het3	Hom1	Hom2	Hom3	Hom4
**DNM3A**	59983	54693	55417	53353	55972	44968	63557	45591	50962	57686	57269
**DNM3L**	54162	89012	113298	37867	22799	32159	49414	13804	11659	16555	19434
**DNMT1**	4919641	5455060	5630842	6884143	5497000	6242194	5053342	3860529	4081037	4724012	4264121
**DPPA3**	94108	101031	129740	145144	63198	79875	99531	69024	72384	70060	98170
**KDM1A**	29601	36365	32424	24382	26902	24211	27475	39516	42139	38617	31596
**KDM1B**	327074	270812	355155	246846	310347	284492	281175	318662	327625	358228	332356
**KDM2B**	50233	51485	51053	71093	61714	67029	60452	33254	32265	39689	30855
**SETD2**	23861	15949	26758	13344	20455	25077	24062	26025	19342	29895	23273
**TIF1B**	124681	152886	120861	96040	97449	91378	123001	91063	97666	86946	83262
**UHRF1**	11675802	9799259	11583398	11091801	9522651	11463501	10829401	7041297	7370016	7605019	9003465
**ZFP57**	63346	58772	85293	90530	79330	80485	69768	82731	62843	78216	61047

* WT1, WT2, Het1, Het2, Hom1, Hom2, etc., denote sample names. WT, wild type or *Nlrp5*^+/+^; Het, *Nlrp5*^*+/*^^−; ^Hom, *Nlrp5*^−/−^.

### DNMT3L and UHRF1 localization altered in *Nlrp5*^−/−^ GV oocytes, while DNMT1 localization was unchanged

**Figure 6.**
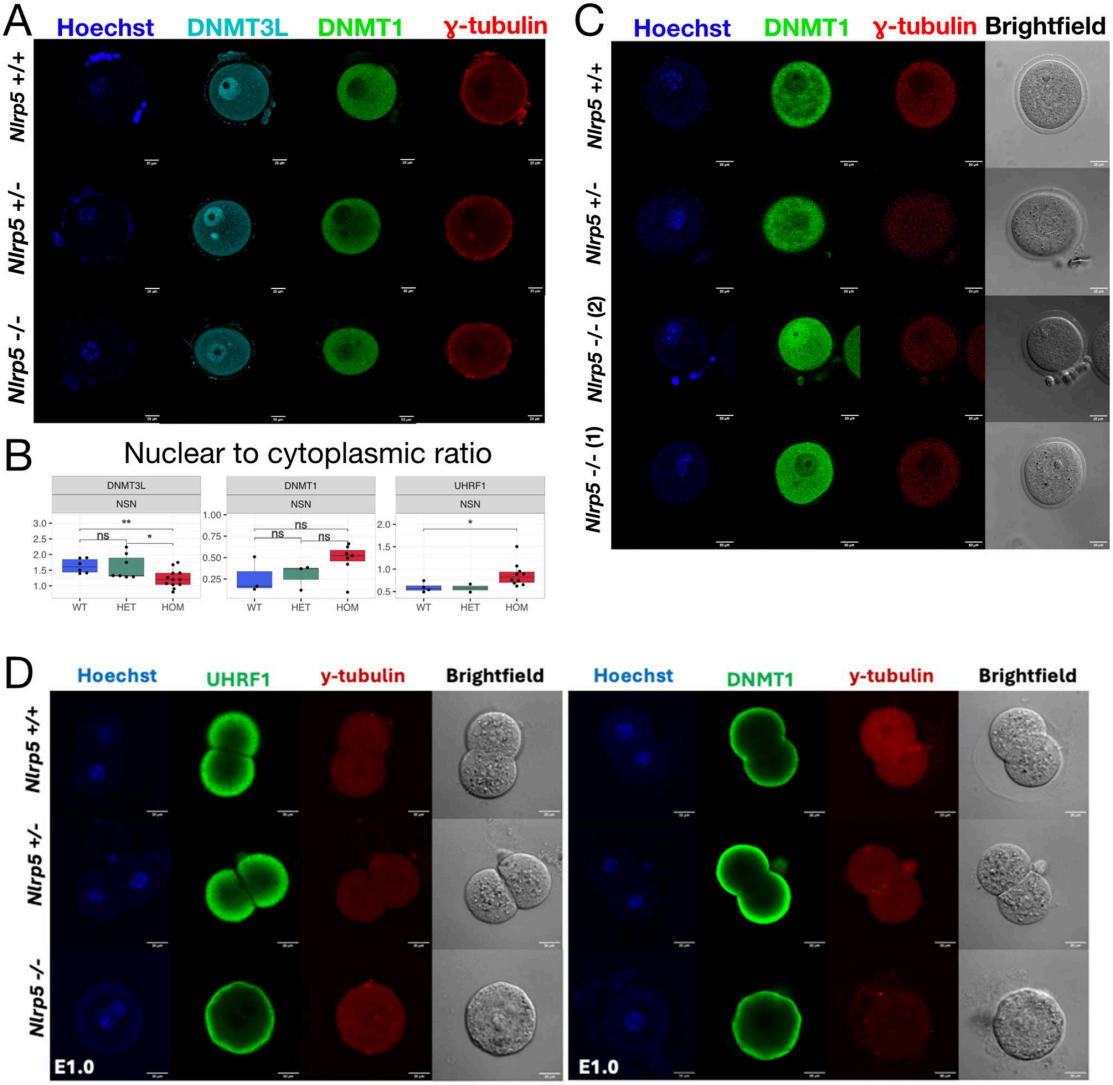
**Immunofluorescence analysis of DNMT3L, DNMT1, and UHRF1 in *Nlrp5*** ^−/−^  **germinal vesicle (GV) oocytes and embryonic day (E)1.0 maternal *Nlrp5***^−/−^  **embryos**. (**A**) Representative immunofluorescence images of non-surrounded nucleolus (NSN)-stage GV *Nlrp5*^−/−^, *Nlrp5^+/^^−^*, and *Nlrp5^+/+^* oocytes with DNMT3L (cyan), DNMT1 (green) and γ-Tubulin (red). Scale bar, 20 µm. (**B**) Box–Whisker plots showing nuclear-to-cytoplasmic fluorescence ratio for DNMT3L (n = 12 *Nlrp5*^−/−^, 7 *Nlrp5^+/^*^−^, 6 *Nlrp5*^+/+^), DNMT1 (n = 7 *Nlrp5*^−/−^, 3 *Nlrp5^+/^*^−^, 3 *Nlrp5*^+/+^), and UHRF1 (n = 10 *Nlrp5*^−/−^, 2 *Nlrp5^+/^*^−^, 4 *Nlrp5*^+/+^) for each genotype. Boxes represent the interquartile range (IQR; 25th–75th percentiles) with the horizontal line indicating the median. Whiskers extend to the most extreme data points within 1.5× IQR from the box; individual points beyond these limits are plotted as outliers. Shapiro–Wilk normality test applied. If *P* > 0.05, Student’s *t*-test was used. If *P* < 0.05, the Wilcoxon rank-sum test was used. Significant at **P < 0.01, *P < 0.05, ns = difference not significant. (**C**) Representative immunofluorescence images of NSN-stage GV *Nlrp5*^−/−^, *Nlrp5^+/^^−^*, and *Nlrp5^+/+^* oocytes with UHRF1 staining (green), γ-Tubulin staining in red and Hoechst DNA staining in blue. All oocytes were collected at 8 weeks old. Scale bar, 20 µm. (**D**) Representative immunofluorescence images of NALP5 maternal ^−/−^, ^*+/*^^−^ and ^+/+^ embryos, at E1.0. Blue, Hoechst DNA staining; green, UHRF1 (left), DNMT1 (right); red, γ-Tubulin. Scale bar, 20 µm.

Due to the finding that DNMT3L is reduced in abundance by over 75% in *Nlrp5*^−/−^ oocytes, we tested whether DNMT3L localization is altered by immunofluorescence. Because of the low numbers of *Nlrp5*^−/−^ SN oocytes, all cross-genotype comparisons were based on staining of NSN oocytes. Relative mean nuclear fluorescence and relative mean cytoplasmic fluorescence of DNMT3L were not significantly different between genotypes (Supplementary Fig. S9). However, when the nuclear-to-cytoplasmic DNMT3L fluorescence ratio was calculated for each oocyte, the ratio was significantly altered in *Nlrp5*^−/−^ oocytes compared to *Nlrp5*^+/+^ and *Nlrp5^+/^*^−^ oocytes (Fig. 6A and B), with relatively less DNMT3L fluorescence in the nucleus.

We also evaluated the localization of DNMT1 and UHRF1, because these proteins have been reported to be mislocalized from the cytoplasm to the nucleus in *Padi6*-null GV oocytes and maternal *Padi6*-KO pre-implantation embryos, while DNMT1 is mislocalized to the nucleus in maternal *Nlrp14*-KO embryos (Yan *et al.*, 2023; Giaccari *et al.*, 2024). There was no significant mislocalization of DNMT1 evident in *Nlrp5*^−/−^ GV oocytes, with DNMT1 staining localized to the cytoplasm in oocytes of all genotypes (Fig. 6A and B, Supplementary Fig. S9). UHRF1 was predominantly cytoplasmic in *Nlrp5^+/^*^−^ and *Nlrp5*^+/+^ oocytes, although some was present in the nucleus. In *Nlrp5*^−/−^ oocytes, however, there was significantly increased nuclear UHRF1 staining (Fig. 6B and C, Supplementary Fig. S9). When assessing embryonic day (E)1.0 embryos generated by IVF from *Nlrp5*^−/−^ oocytes, we found that neither DNMT1 nor UHRF1 was altered in localization compared with wild-type E1.0 embryos (Fig. 6D). Note, however, smaller differences may be obscured by differences in embryo staging between the maternal *Nlrp5*-KO embryos and controls. This misregulation of epigenetic modifier proteins in mouse SCMC mutants, including in *Nlrp5*, provides a biological mechanism for the methylation and imprinting phenotypes seen *in vivo*.

### Low level of global DNA hypomethylation in *Nlrp5*^−/−^ GV oocytes coinciding with hypomethylation over a subset of gDMRs

**Figure 7.**
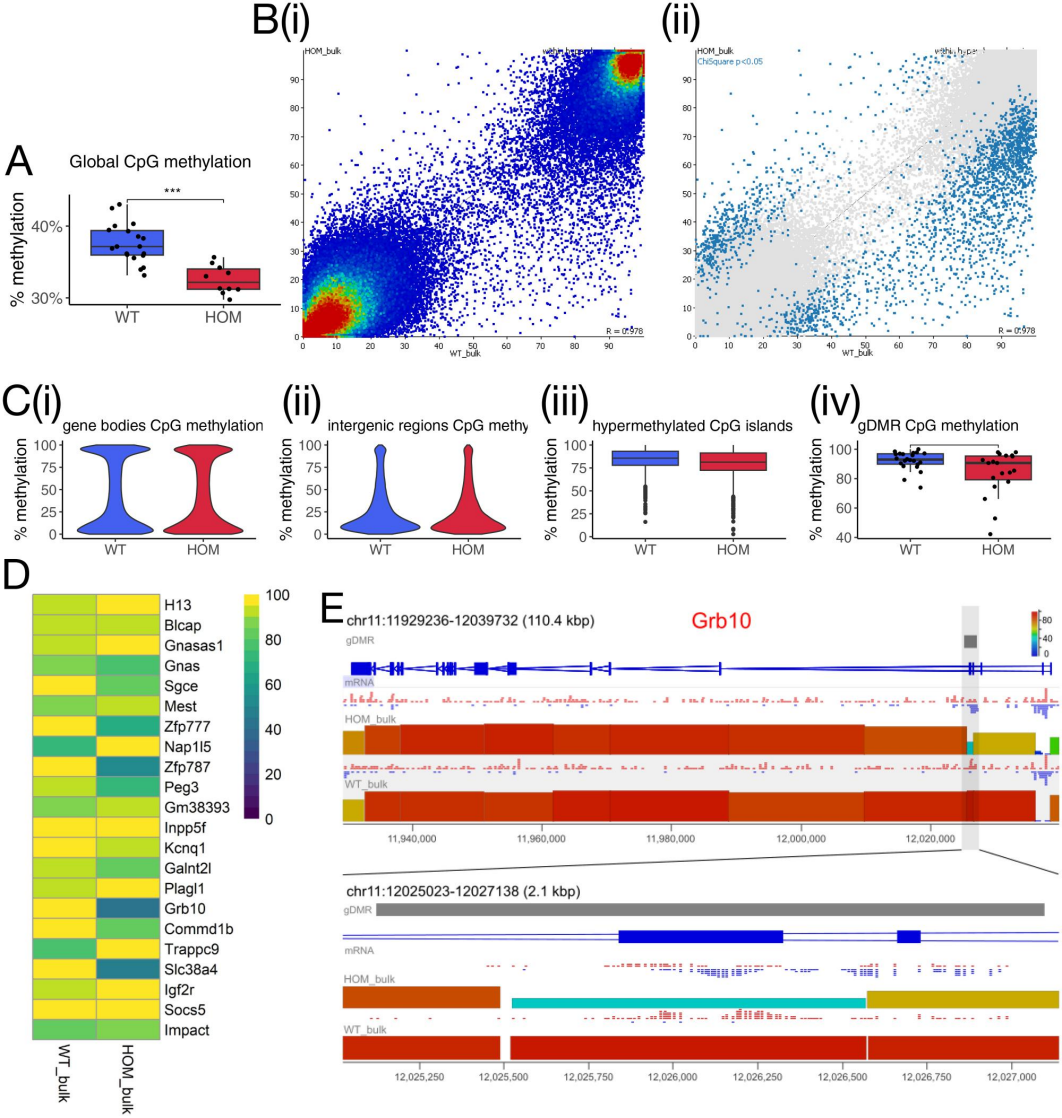
**Global attenuation of DNA methylation overlapping with germline differentially methylated regions (gDMRs)**. (**A**) Box–Whisker plot showing global % CpG methylation of each WT (*Nlrp5*^+/+^) and HOM (*Nlrp5*^−/−^) oocyte, determined as the mean % methylation of all informative 100-CpG tiles (231 959). Boxes represent the interquartile range (IQR; 25th–75th percentiles) with the horizontal line indicating the median. Whiskers extend to the most extreme data points within 1.5× IQR from the box; individual points beyond these limits are plotted as outliers. Welch *t*-test, ***P < 0.001. (**B**) (i) Scatterplot of bulked *Nlrp5*^+/+^ (WT, *x*-axis) and *Nlrp5*^−/−^ (HOM, *y*-axis) samples displaying all 100-CpG tiles within hypo- and hypermethylated domains (152 071 tiles), coloured for density. (ii) Scatterplot of tiles called differentially methylated (tiles with chi-square *P*-value of <0.05; 2452 tiles). (**C**) Violin and Box–Whisker plots showing % methylation over certain genomic features: (i) Gene body cytosine-guanine dinucleotide (CpG) methylation (152 071 tiles), (ii) intergenic region CpG methylation (68,800 tiles), (iii) annotated hypermethylated CpG islands (1796), and (iv) gDMR CpG methylation (each dot represents the group mean % methylation of a given gDMR), Wilcoxon rank-sum test showing borderline but not significant *P*-value (significance cut-off <= 0.05). Boxes represent the interquartile range (IQR; 25th–75th percentiles) with the horizontal line indicating the median. Whiskers extend to the most extreme data points within 1.5× IQR from the box; individual points beyond these limits are plotted as outliers. (**D**) Heatmap showing % methylation at listed oocyte gDMRs in *Nlrp5*^−/−^ (HOM) and *Nlrp5*^+/+^ (WT) pseudo-bulked samples. (**E**) Example of hypomethylated gDMR: SeqMonk genome browser view of the *Grb10* locus in *Nlrp5*^−/−^ and *Nlrp5*^+/+^ datasets, with (below) zoom-in on the *Grb10* gDMR. Each coloured block represents a 100-CpG tile, with height and colour-coding indicating % methylation of CpGs called across the tile. Methylation calls of individual CpG sites are indicated above as red (methylated) or blue (unmethylated) ticks.

To understand the epigenetic effects of the absence of NALP5, GV-stage oocytes from *Nlrp5*^−/−^ and *Nlrp5*^+/+^ females were processed via scPBAT-seq to generate libraries for whole-genome DNA methylation analysis (Macaulay *et al.*, 2015; Clark *et al.*, 2017). Because DNMT3L is essential for *de novo* methylation in mouse oocytes (Smallwood *et al.*, 2011; Shirane *et al.*, 2013), the significant reduction of DNMT3L in *Nlrp5*^−/−^ oocytes identified by mass spectrometry suggested the possibility of impaired methylation. After filtering out samples with low read counts or suspected somatic cell DNA contamination, 10 *Nlrp5*^−/−^ oocytes and 19 *Nlrp5*^+/+^ oocytes remained for analysis (Supplementary File S3 (tab i)). Methylation was quantitated across 100-CpG consecutive tiles in the genome. The average global CpG methylation percentage was significantly lower (*P*-value <0.001) in the *Nlrp5*^−/−^ compared to *Nlrp5*^+/+^ oocytes, with a difference of 4.98% between median global CpG methylation in *Nlrp5*^−/−^ and *Nlrp5*^+/+^ samples (Fig. 7A). For further analysis, samples were pseudo-bulked for higher coverage of gene features. The *Nlrp5*^−/−^ pseudo-bulked cohort had a combined total of 31.6 million reads, and the *Nlrp5*^+/+^ pseudo-bulked cohort had a total of 28.2 million reads.

*Nlrp5* ^−/−^ and *Nlrp5*^+/+^ samples recapitulated the typical bimodal methylation patterning unique to oocytes (Demond and Kelsey, 2020) (Fig. 7B), and percentage methylation at gene bodies, intergenic regions, and annotated hypermethylated CpG islands (CGIs) was not significantly different between *Nlrp5*^−/−^ and *Nlrp5*^+/+^ oocytes (Fig. 7C). To test for sites of differential methylation, a chi-square test was performed on 100-CpG tiles within annotated oocyte hyper- and hypomethylated domains (Veselovska *et al.*, 2015) (significance <0.05 after multiple testing correction, minimum percentage difference = 25%). The test produced 2452 differentially methylated hits between the *Nlrp5*^−/−^ and *Nlrp5*^+/+^ groups (Fig. 7B(ii)). When assessing methylation at all annotated oocyte gDMRs, there was no significant difference between genotypes across all gDMRs (Fig. 7C(iv)). However, 3 individual gDMRs (*Zfp777*, *Zfp787*, and *Grb10*; Fig. 7D and E) were significantly hypomethylated in the *Nlrp5*^−/−^ group compared to *Nlrp5*^+/+^ oocyte probes, as determined by chi-square test on 100-CpG tiles overlapping with gDMRs (Supplementary File S3 (tab ii)).

To confirm that the observed global hypomethylation in the *Nlrp5*^−/−^ oocytes could not be attributed to a skew in the NSN–SN staging ratio, a comparative analysis was performed on a wild-type NSN/SN mouse GV oocyte staging methylation dataset (Castillo-Fernandez *et al.*, 2020). The staging data were quantitated using the same 100-CpG tiles across the genome (Supplementary File S3 (tab iii)). When PCA was performed on samples from the *Nlrp5* and staging datasets, *Nlrp5*^−/−^ samples separated out from all wild-type samples, including those of the SN/NSN staging dataset. Although there was some effect of staging, there was limited overlap of *Nlrp5*^−/−^ samples with NSN samples (Supplementary Fig. S10). Furthermore, none of the three gDMRs identified as differentially methylated in *Nlrp5*^−/−^ GV oocytes were significantly differentially methylated in the staging dataset.

## Discussion

Multi-omic analysis of GV oocytes from our CRISPR/Cas9-generated *Nlrp5*^−/−^ mice demonstrates that SCMC integrity is lost, making *Nlrp5* mutants an ideal model in which to study overall SCMC function. We find that in *Nlrp5*^−/−^ GV oocytes, the abundance and/or localization of some key epigenetic factors is altered, and there is a modest global hypomethylation, which encompasses some gDMRs. Additionally, the ratio of NSN–SN stage oocytes in the *Nlrp5*^−/−^ cohort skews towards the less mature NSN stage, detectable as early as 3 weeks old. While this staging skew likely contributes to some of the methylation differences observed, we show that it does not account for all of the differences in DNA methylation. Indeed, the three significantly hypomethylated gDMRs detected in *Nlrp5*^−/−^ GV oocytes are not significantly hypomethylated in wild-type NSN GV oocytes. Immunofluorescence staining of GV oocytes showed that, while NALP5 was ablated in *Nlrp5*^−/−^ GV oocytes, it was present throughout the cytoplasm in *Nlrp5^+/^*^−^ and *Nlrp5*^+/+^ oocytes, not just at the subcortex, supporting recent studies describing the presence of SCMC proteins throughout the cytoplasm (Jentoft *et al.*, 2023). *Nlrp5^+/^*^−^ oocytes lacked subcortical saturation of NALP5 fluorescence. Given that *Nlrp5^+/^*^−^ oocytes are developmentally competent, the previously described subcortical localization of NALP5 (and potentially other SCMC proteins) in wild-type oocytes is unlikely to be biologically relevant.

### Two functional subcategories of SCMC proteins exist

A comparative analysis with *Tle6*-KO oocytes (Jentoft *et al.*, 2023) demonstrated the same trends in reduction of SCMC proteins as in *Nlrp5*^−/−^ oocytes, suggesting similar effects of these mutants on integrity of a subset of SCMC proteins. Three known SCMC proteins were not altered in abundance in the *Nlrp5*^−/−^ oocytes: PADI6, NLRP14, and NLRP2. These three SCMC proteins were also unaltered in the *Tle6* KO. Comparative analysis with *Padi6* KO oocytes (Jentoft *et al.*, 2023) demonstrated the opposite trends: reductions in NLRP14 and NLRP2, but little to no effect on other SCMC proteins. These trends indicate that two functionally separate subgroups of SCMC proteins exist, the NALP5–TLE6 group, and the PADI6-dependent group. This is further supported by the global comparative analysis of these three datasets (*Nlrp5-*, *Tle6-*, *Padi6*-nulls), which shows a greater overall similarity between *Nlrp5*^−/−^ and *Tle6*^−/−^ oocyte proteomes than either shares with *Padi6*^−/−^ oocytes. Two new candidates for SCMC membership were identified in the *Nlrp5*^−/−^ data, NLRP4b and NLRP9a, both of which were significantly reduced in *Nlrp5*^−/−^ oocytes. Various proteins of the reproductive clade of the NLRP family (Monk *et al.*, 2017) appear to interact with other SCMC proteins with a degree of redundancy, as their abundances differ greatly, even between *Nlrp5* and *Tle6* KOs.

Previous studies have suggested that the SCMC may regulate the stability and availability of maternal proteins, potentially facilitating their accessibility to nuclear translocation factors at key timepoints in development (Yurttas *et al.*, 2008; Kim *et al.*, 2010; Tashiro *et al.*, 2010; Jentoft *et al.*, 2023). By investigating the overlapping roles of the SCMC and CPLs, the studies have argued that this may not only ensure the timely and efficient localization of epigenetic modifiers (and other regulatory factors) to the nucleus, but may also protect them against their premature degradation by the proteasome. Indeed, the SCMC may act as a structural scaffold for storage and stabilization of maternal factors necessary for development, and the presence of organized CPLs may simply reflect a functional SCMC.

In the present study, a subset of SCMC proteins, as well as three epigenetic modifiers, DNMT3L, UHRF1, and KDM2B, and a broad range of other proteins important for oocyte development, were significantly altered in abundance, reflecting a strong effect of the KO on the *Nlrp5*^−/−^ oocyte proteome. In contrast, there were no effects on SCMC or listed epigenetic modifier genes observable in the *Nlrp5*^−/−^ transcriptome, and the overall correlation of DAPs with DEGs was low. While a level of misregulation is observable in both the proteome and transcriptome, the proteome shows a larger effect, and more specific changes to oocyte maternally stored factors. This indicates that a greater level of misregulation occurs post-translationally, and that the eventual two-cell arrest phenotype observed in embryos derived from *Nlrp5*^−/−^ oocytes is likely a result of deficiencies in maternally stored proteins, arising from ineffective storage or localization at key developmental timepoints. The *Nlrp5* null mutation affects the abundance of a wide variety of different proteins, from building blocks of the cytoskeleton and modifiers of heterochromatin to factors involved in protein transport between the ER and the Golgi, in nuclear transport, and in epigenetic modification. This variety of regulatory targets suggests a broad role of NALP5 and the SCMC in storage and sequestration of maternal factors in the oocyte, binding a wide variety of proteins to protect them from proteasomal degradation, premature assembly into organelles or functional complexes, or premature translocation into the nucleus. This is especially important in the oocyte, where, after transcriptional arrest at the SN GV stage, further oocyte development and early embryo development are entirely mediated by the pre-existing pool of maternal factors.

The differences in effects on the proteome in *Nlrp5*-, *Tle6*-, and *Padi6*-null oocytes suggest that some groups of proteins are preferentially bound by certain SCMC subgroups, whereas others may be specifically regulated by individual SCMC proteins. Similarities in downregulated tubulin-related proteins in *Padi6* and *Nlrp14*-nulls suggest that this subgroup of the SCMC has a shared function in microtubule-regulation independent of NALP5 and TLE6, although NALP5, independently from TLE6, seems to regulate the cytoskeleton via TBAL3 and TAGL2. UHRF1 appears to be preferentially regulated by the PADI6–NLRP14 subgroup, with stronger reductions of UHRF1 abundance in these mutants than in *Nlrp5* KOs, although this and another recent study show interactions between NALP5 and UHRF1 (Unoki *et al.*, 2024). Other DAPs identified in the *Nlrp5*^−/−^ samples, such as FABP3, ERGIC1, SATB1, GDF9, and BMP15, were not significantly altered in abundance in the other SCMC KO datasets, indicating that they are preferentially regulated by NALP5. FABP3 is a key factor in lipid metabolism and protection against lipid toxicity (Lee *et al.*, 2020). ERGIC1 is a cycling membrane protein with roles in protein transport between the endoplasmic reticulum and Golgi (Breuza *et al.*, 2004). SATB1 is a chromatin organizer and transcription factor that regulates several cellular processes, such as differentiation, proliferation, and apoptosis (Sunkara *et al.*, 2018). GDF9 and BMP15 are both important in folliculogenesis and the regulation of ovulation. These are just a few of the proteins significantly altered in abundance in *Nlrp5*^−/−^ oocytes, illustrating the variety of proteins and pathways potentially regulated by the SCMC and NALP5 individually.

### Roles for NALP5 in DNA methylation and genomic imprinting

Three epigenetic modifier proteins, DNMT3L, UHRF1, and KDM2B, were significantly reduced in *Nlrp5*^−/−^ oocytes; *Nlrp5*^−/−^ oocytes also had proportionally less DNMT3L in their nucleus than cytoplasm, and proportionately more UHRF1 in their nuclei, suggesting that NALP5 affects DNMT3L and UHRF1 localization as well as abundance. *Dnmt3l*-null female mice are viable but sterile, with offspring lethality attributed to abnormal maternal imprinting (Bourc’his and Bestor, 2004). A 75% reduction in oocyte DNMT3L could affect *de novo* methylation via its influence on the catalytic activity of DNMT3A (Smallwood and Kelsey, 2012; Shirane *et al.*, 2013), and levels of global DNA methylation would be expected to be lower than in wild-type GV oocytes. While the mild 30% reduction in total UHRF1 abundance in *Nlrp5*^−/−^ oocytes would not suggest a biological effect on UHRF1-mediated *de novo* methylation, the mislocalization of the UHRF1 to the nucleus in these oocytes means that an effect of UHRF1 on levels of DNA methylation in these oocytes cannot be ruled out. One hypothesis is that a nuclear reduction of DNMT3L could be partially supplemented by a nuclear increase in UHRF1, resulting in a more subtle hypomethylation phenotype. Indeed, *Nlrp5*^−/−^ oocytes showed a low but significant level of global hypomethylation that could not be explained purely by potential staging differences between *Nlrp5*^−/−^ and *Nlrp5*^+/+^ samples. This modest global hypomethylation may be due to an attenuation or delay in complete *de novo* DNA methylation as a result of low DNMT3L availability. In addition to the global hypomethylation observed in *Nlrp5*^−/−^ oocytes, several significantly hypomethylated regions were identified, including three that coincided with known imprinted gDMRs or that shared a promoter with a nearby gDMR. The limited number of gDMRs affected would suggest that these are the DMRs most susceptible to deficiencies in the *de novo* methylation apparatus.

While UHRF1 and DNMT1 can contribute to *de novo* methylation in oocytes (Shirane *et al.*, 2013; Maenohara *et al.*, 2017), and UHRF1 may have roles independent of UHRF1 (Maenohara *et al.*, 2017), they are particularly crucial for maintenance of DNA methylation. UHRF1 was the most strongly reduced protein in *Padi6* and *Nlrp14* nulls (∼90%), aside from PADI6 and NLRP14, respectively. Furthermore, UHRF1 and DNMT1 were mislocalized to the nucleus in MII *Padi6*-null oocytes and maternal *Padi6*-null embryos, coinciding with mild genomic hypermethylation in the oocytes and a more dramatic hypermethylation in the embryos, likely due to excessive maintenance of DNA methylation marks and failure of demethylation. Similarly, in *Nlrp14*^−/−^ oocytes, UHRF1 was mislocalized to the nucleus (DNMT1 localization was not studied), although the methylation was not significantly altered. While UHRF1 was also mildly reduced (∼30%) in *Nlrp5*^−/−^ GV oocytes, it is not known to be haploinsufficient and thus, taken together with there being no significant effect on DNMT1 abundance or localization in the *Nlrp5*^−/−^ GV oocytes, we think it is unlikely to account for the hypomethylation we observe. But given the interactions of UHRF1 with components of either form of the SCMC (Uemura *et al.*, 2023; Unoki *et al.*, 2024), the reduction could lead to a complexity of impairments in the oocyte, with a particular impact post-fertilization (Maenohara *et al.*, 2017). Thus, NALP5 appears preferentially to contribute to *de novo* methylation, while PADI6 seems instead preferentially to be required for methylation maintenance, indicating a balancing role of the two SCMC subgroups in maintaining normal DNA methylation patterning, via regulation and timely localization of either *de novo* DNA methylation establishment proteins or maintenance factors.

KDM2B is an H3 demethylase known to target H3K4me3 and H3K36me2. It is part of the variant PRC1 complex, recruiting Polycomb-1 proteins to unmethylated CpGs (Farcas *et al.*, 2012; Dimitrova *et al.*, 2015). It was significantly reduced, by over 40%, in *Nlrp5*^−/−^ oocytes and significantly reduced, by ∼30%, in *Padi6*^−/−^ oocytes. The significant reduction of this protein in the *Nlrp5*^−/−^ oocytes may influence epigenetic reprogramming indirectly by causing a lower level of Polycomb-1 protein recruitment to unmethylated CpG regions. Interestingly, however, an oocyte-specific *Kdm2b* KO does not adversely affect embryonic development (Nishimura *et al.*, 2023), so the biological relevance of KDM2B in oocytes remains to be determined. Another histone demethylase, KDM1B, was significantly increased in abundance in the *Padi6-*null dataset, and listed as significantly increased in *Nlrp14*^−/−^ oocytes (Zhang *et al.*, 2023), but was not altered in *Nlrp5* or *Tle6*-nulls. Oocytes from KDM1B-deficient females show loss of methylation at CGIs, including most imprinted gDMRs (Ciccone *et al.*, 2009; Stewart *et al.*, 2015), and embryos derived from *Kdm1b*^−/−^ oocytes die before mid-gestation with imprinted gene deregulation (Ciccone *et al.*, 2009). The increased abundance of KDM1B exclusively in *Padi6-* and *Nlrp14*-nulls may indicate a separate role of the PADI6-NLRP14 SCMC subset in epigenetic regulation.

The epigenetic modifier proteins altered in abundance in *Nlrp5*^−/−^ oocytes demonstrate that a mutation in one core SCMC gene can potentially mis-regulate *de novo* establishment of DNA methylation (via DNMT3L and potentially UHRF1) in the mouse oocyte, DNA methylation maintenance (via UHRF1) in the pre-implantation embryo, or histone modifications (via KDM2B) depending on the degree of altered protein abundance or mislocalization. The downstream effects of SCMC mutations on DNA methylation in the embryo or later development are likely to be determined by the composition of the repertoire of proteins misregulated by a given SCMC mutation, to varying degrees of penetrance depending on severity of mutation and the redundancies between some SCMC components. It seems unlikely that the ∼5% reduction in global methylation in *Nlrp5*^−/−^ oocytes that we observe will have an effect on early embryo development, especially given the genome-wide methylation reprogramming in the zygote. But a focal reduction in methylation of a gDMR could be more meaningful, if it involved a gDMR for an imprinted gene of developmental significance like *Grb10*, and occurred over features of the gDMR such as ZFP57/ZNF445 binding sites (Juan and Bartolomei, 2019) at which methylation is critical for maintenance of imprinting and appropriate expression of the associated gene in the embryo.

These findings have implications for SCMC defects in humans, where severe global hypomethylation has been reported in oocytes deficient in the SCMC component KHDC3L (Demond *et al.*, 2019). The substantial deficiency in DNMT3L we observe in *Nlrp5*^−/−^ mouse GV oocytes provides a plausible mechanism for the modest global hypomethylation we observe, including at susceptible imprinted regions. However, the mouse model does not satisfactorily explain the human phenotype, as DNMT3L is not expressed in human oocytes (Petrussa *et al.*, 2014). While DNMT3L acts as a critical cofactor for DNMT3A-mediated *de novo* methylation in mouse oocytes, recent data indicate that alternative molecular mechanisms must compensate for the absence of DNMT3L expression during oogenesis in humans (Behluli *et al.*, 2023). The precise identity of these mechanisms remains poorly understood but may involve other DNA methyltransferase paralogs, regulatory factors, or chromatin environment features that can establish methylation imprints independently of DNMT3L. Therefore, while this work supports an important link between the SCMC and DNA methylation patterning via the stability and localization of epigenetic modifiers, the varied and species-specific effects of each individual SCMC subunit (Giaccari *et al.*, 2024) mean that specific human phenotypes will have to be further explored in models more similar to humans.

## Conclusion

This study points towards a mechanistic explanation for the link between SCMC integrity in the oocyte and faithful DNA methylation patterning. Through multi-omic profiling of *Nlrp5*^−/−^ and^*+/*^^−^GV oocytes, coupled with comparative proteomics analysis with other SCMC mutants, we consider how mutations in the SCMC can lead to a plethora of different imprinting and DNA methylation disorders, depending on the SCMC component in question, and on the penetrance of the SCMC mutation. We demonstrate that the NALP5 mutant phenotype is a consequence of misregulation of stability of a variety of oocyte maternal factors, with detrimental effects on several different biological processes crucial to oocyte developmental competence. One of the processes affected is *de novo* DNA methylation establishment, and a low level of global hypomethylation is observed in the *Nlrp5*^−/−^ oocyte genome, overlapping with DMRs coinciding with three known imprinted genes. This is likely due to the adverse effects of the NALP5 ablation on the abundance and localization of DNMT3L, a key cofactor for murine *de novo* DNA methylation.

## Supplementary Material

gaaf055_Supplementary_Data

## Data Availability

Single-cell RNA and PBAT sequencing data generated during this study have been deposited at Gene Expression Omnibus database (https://www.ncbi.nlm.nih.gov/geo/) and are available as of the date of publication under accession number: GSE278144. Mass spectrometry (Orbitrap Eclipse, Thermo Fisher Scientific) data have been deposited to the ProteomicXchange Consortium via the PRIDE partner repository (https://www.ebi.ac.uk/pride/) with the dataset identifier PXD056859, available as of the date of publication. This paper does not report original code. Further information and requests for resources and reagents should be directed to Gavin Kelsey (gavin.kelsey@babraham.ac.uk).
