# Supplementary material for: An Nlrp5-null mutation leads to attenuated de novo methylation in oocytes, accompanied by a significant reduction in DNMT3L
Source: Mol Hum Reprod. 2025 Nov 18;31(4):gaaf055. doi: 10.1093/molehr/gaaf055 (PMC12704423; doi:10.1093/molehr/gaaf055)
Supplement: gaaf055_Supplementary_Data [file gaaf055_supplementary_data.zip › Supplementary_information_EO.pdf]

An *Nlrp5*-null mutation leads to attenuated *de novo* methylation in oocytes, accompanied by a significant reduction in DNMT3L.

LEAH NIC AODHA[1,2], ALEXANDRA POKHILKO[1], LEAH U ROSEN[3,4], STYLIANI GALATIDOU[5,6], EDYTA WALEWSKA[1], CHRISTIAN BELTON[1,7], ANTONIO GALVAO[8], HANNEKE OKKENHAUG[9], LU YU[10], ASIF NAKHUDA[11], BILL MANSFIELD[12], SOUMEN KHAN[1], DAVID OXLEY[10], MONTSERRAT BARRAGÁN[5], GAVIN KELSEY[1,7,13] *

Supplementary figures:

Supplementary Figure S1 – Oocyte staging heatmap.

Supplementary Figure S2 – RNA-sequencing quality control (QC) plots.

Supplementary Figure S3 – Single cell RNA-sequencing MA plots.

Supplementary Fgure S4 – *Nlrp5 +/-* RNA-sequencing volcano plots.

Supplementary Figure S5 – Enrichment analysis of RNA-seq DEGs in Germinal Vesicle stage *Nlrp5 -/-* oocytes.

Supplementary Figure S6 – Hierarchical clustering of raw protein abundance values between bulk germinal vesicle stage oocyte samples of the three genotypes.

Supplementary Figure S7 – Violin plot showing the $\log_2$ fold change of each protein detected in the *Nlrp5 -/-* oocyte proteomics data.

Supplementary Figure S8 – Enrichment analysis of differentially abundant proteins in *Nlrp5 -/-* samples.

Supplementary Figure S9 – Mean nuclear and cytoplasmic fluorescence values for non-surrounded nucleolus germinal vesicle stage oocytes of each genotype.

Supplementary Figure S10 – ScPBAT-sequencing PCA embeddings of GV-stage oocytes from the present study and the NSN-SN staging dataset.

Supplementary Files (provided as separate Excel files):

Supplementary File S1 – Transcriptomics (scRNA-seq).

S1(tab i) Marker genes for oocyte staging.

S1(tab ii) *Nlrp5 -/-* (Hom) vs *Nlrp5 +/+* (WT) differentially expressed genes.

S1(tab iii) *Nlrp5 +/-* (Het) vs *Nlrp5 +/+* (WT) differentially expressed genes.

Supplementary File S2 – Proteomics (mass spectrometry).

S2(tab i) Raw protein abundance counts for all samples.

S2(tab ii) Log-normalised protein abundance counts for all samples.

S2(tab iii) *Nlrp5 -/-* (Hom) vs *Nlrp5 +/+* (WT) differentially abundant proteins.

S2(tab iv) *Nlrp5 +/-* (Het) vs *Nlrp5 +/+* (WT) differentially abundant proteins.

S2(tab v) *Padi6 +/-* (Het) vs *Padi6 -/-* (Hom) differentially abundant proteins.

S2(tab vi) *Tle6 +/-* (Het) vs *Tle6 -/-* (Hom) differentially abundant proteins.

Supplementary File S3 – DNA methylation (scPBAT-seq).

S3(tab i) Single cell PBAT quality control metrics for each sample.

S3(tab ii) *Nlrp5 -/-* (Hom) vs *Nlrp5 +/+* (WT) germline differentially methylated regions.

S3(tab iii) Germline differentially methylated regions in non-surrounded nucleolus (NSN) vs surrounded nucleolus SN oocyte samples.

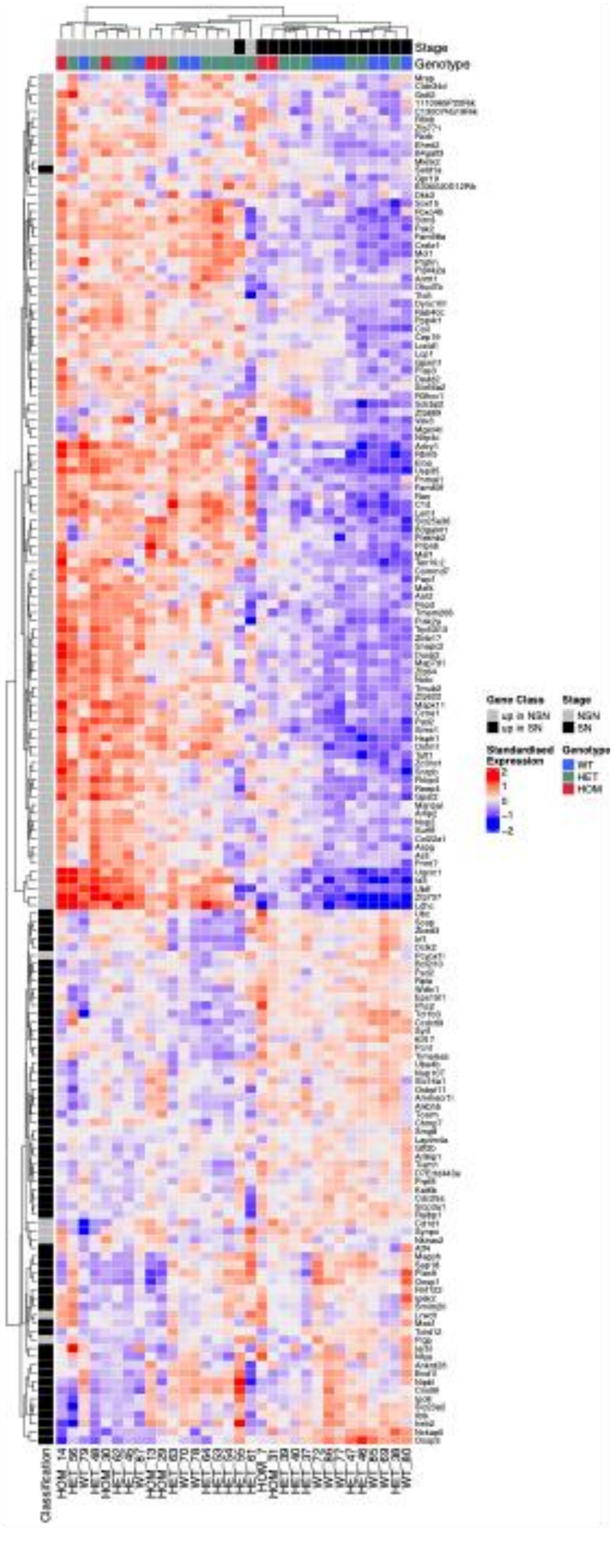

Supplementary Figure S1: Oocyte staging heatmap. Heatmaps showing classification of scRNA-seq oocyte samples as either Non-surrounded nucleolus (NSN) or Surrounded nucleolus (SN) based on expression of marker genes for each stage (Gene list in Supplementary File S1). Staging based on marker gene expression in *Nlrp5 -/-, Nlrp5 +/-,* and wild type Germinal Vesicle oocyte samples. Stage (NSN = grey, SN = black) and genotype (red = *Nlrp5 -/-* (HOM), green = *Nlrp5 +/-* (HET), and blue = wild type (WT)) denoted by bars along the top of the heatmap. Only samples that passed read count QC and that could be clearly classified using this gene list were included in these heat maps.

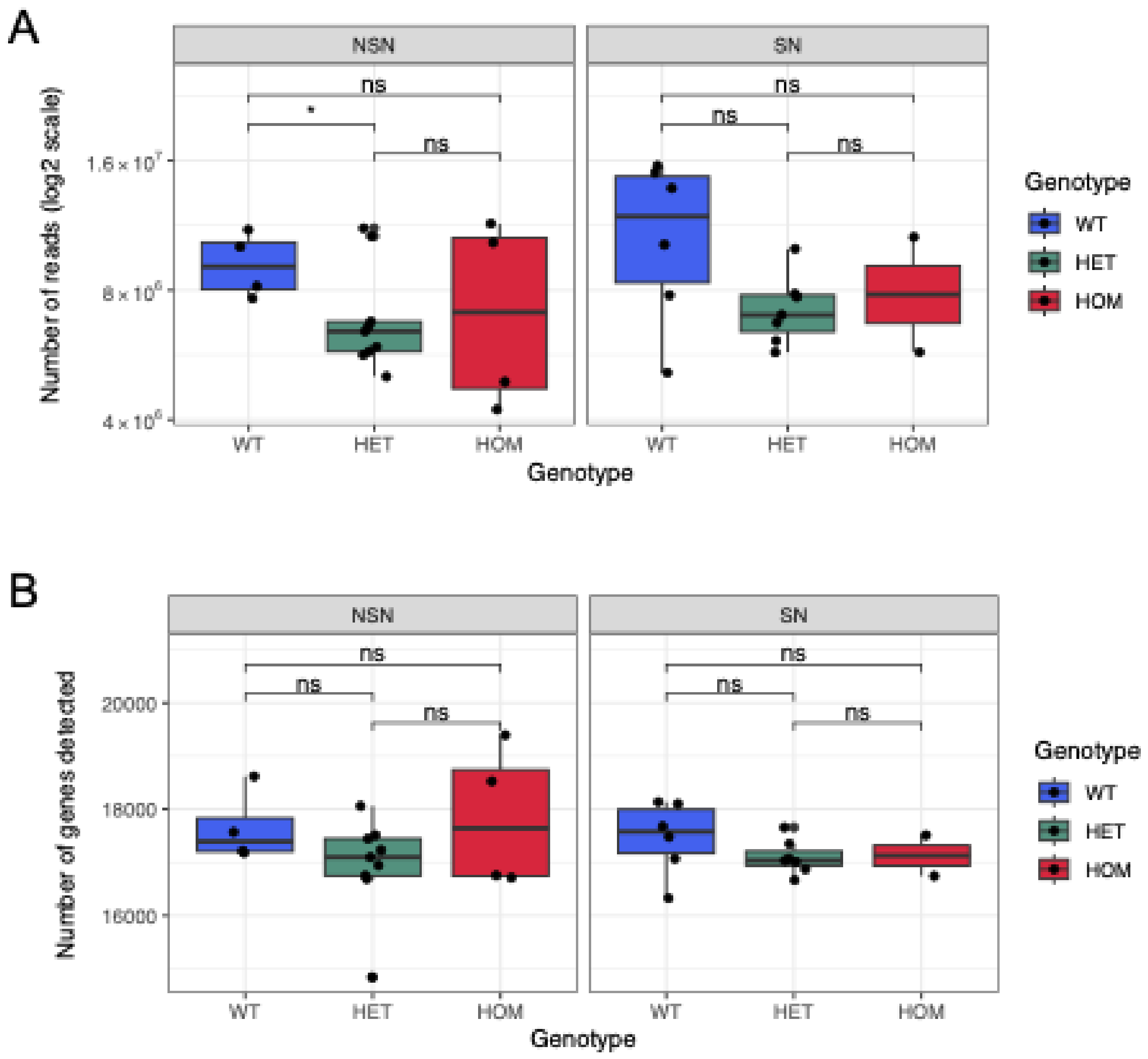

Supplementary Figure S2: RNA-sequencing quality control (QC) plots. RNA-seq QC plots showing **(A)** total read count per sample, Log_2 scale (t-test for significance, but on the log_2 of the number of reads to normalize the data. * p= <0.05, ns = not significant), and **(B)** number of genes detected per sample (t-test for significance: p= <0.05, ns = not significant), separated by non-surrounded nucleolus (NSN)/ surrounded nucleolus (SN) stage and genotype (HOM = *Nlrp5 -/-,* HET = *Nlrp5 +/-,* WT = Wild type) . Number of samples categorised as 'NSN' or 'SN' per genotype = 4 NSN *Nlrp5 -/-,* 2 SN *Nlrp5 -/-,* 4 NSN *Nlrp5 +/+,* 6 SN *Nlrp5 +/+.*

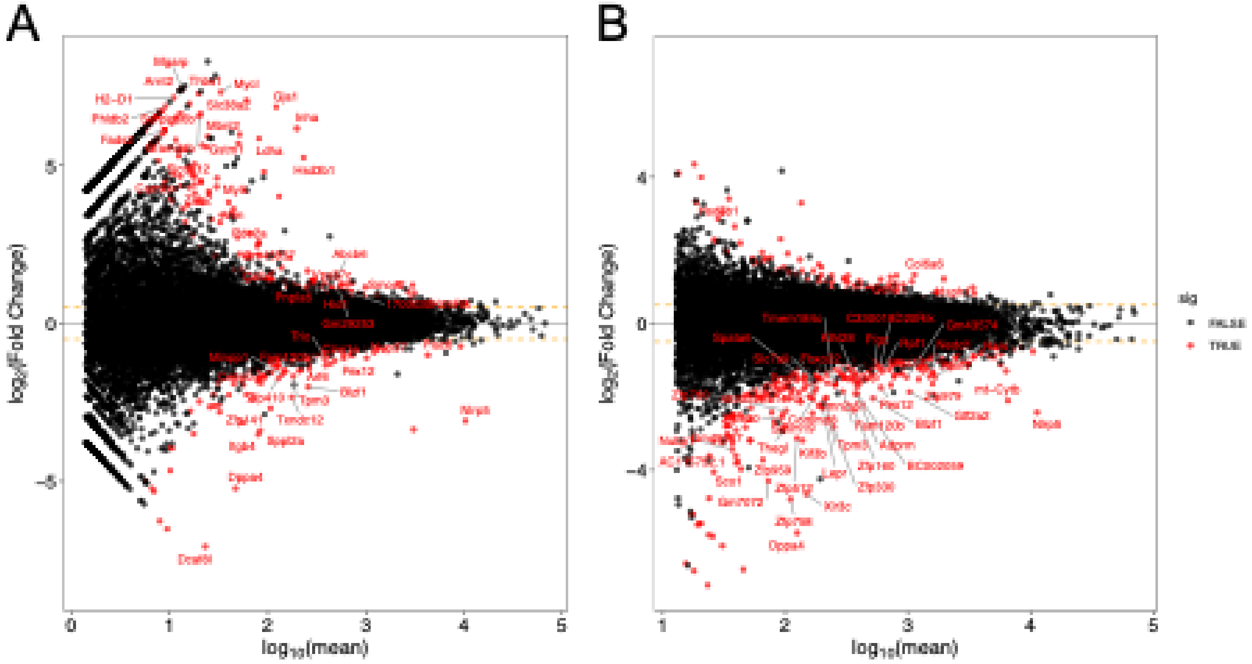

Supplementary Figure S3: Single cell RNA-sequencing MA plots. scRNA-seq MA plots showing changes in gene expression for *Nlrp5 -/-* vs wild-type (*Nlrp5 +/+*) comparison*.* MA plot for **(A)** Non-Surrounded Nucleolus(NSN)-stage scRNA-seq oocyte library samples and **(B)** for Surrounded Nucleolus(SN)-stage scRNA-seq oocyte library samples. The log_2 fold changes in mean expression in *Nlrp5 -/-* samples relative to wild-type samples is shown against log_{10} of the mean expression. Red dots indicate DEGs (log2 FC <-0.5 or >0.5, BH-adjusted p value <0.05).

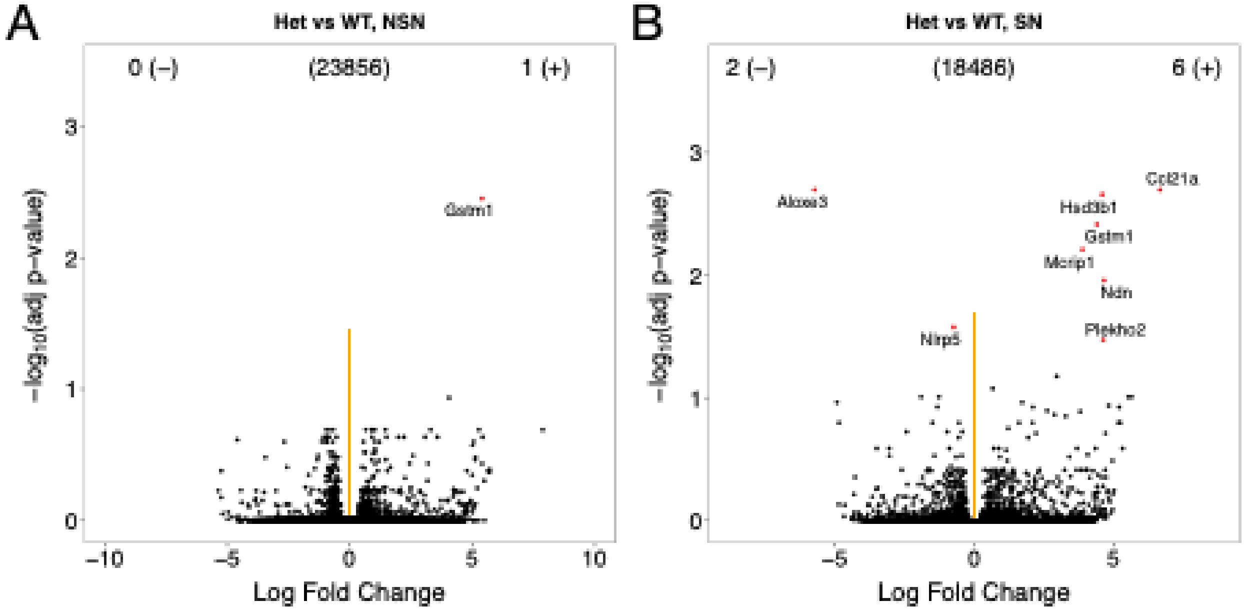

Supplementary Figure S4: *Nlrp5 +/-* RNA-sequencing volcano plots. Volcano plots showing *Nlrp5 +/-* DEGs in the *Nlrp5 +/-* vs *Nlrp5 +/+* (WT) comparison, at **(A)** non-surrounded nucleolus (NSN) and **(B)** surrounded nucleolus (SN) stage. Significant DEGs highlighted in red (significant where log2 fold change is <-0.5 or >0.5, and BH-adjusted p value is <0.05).

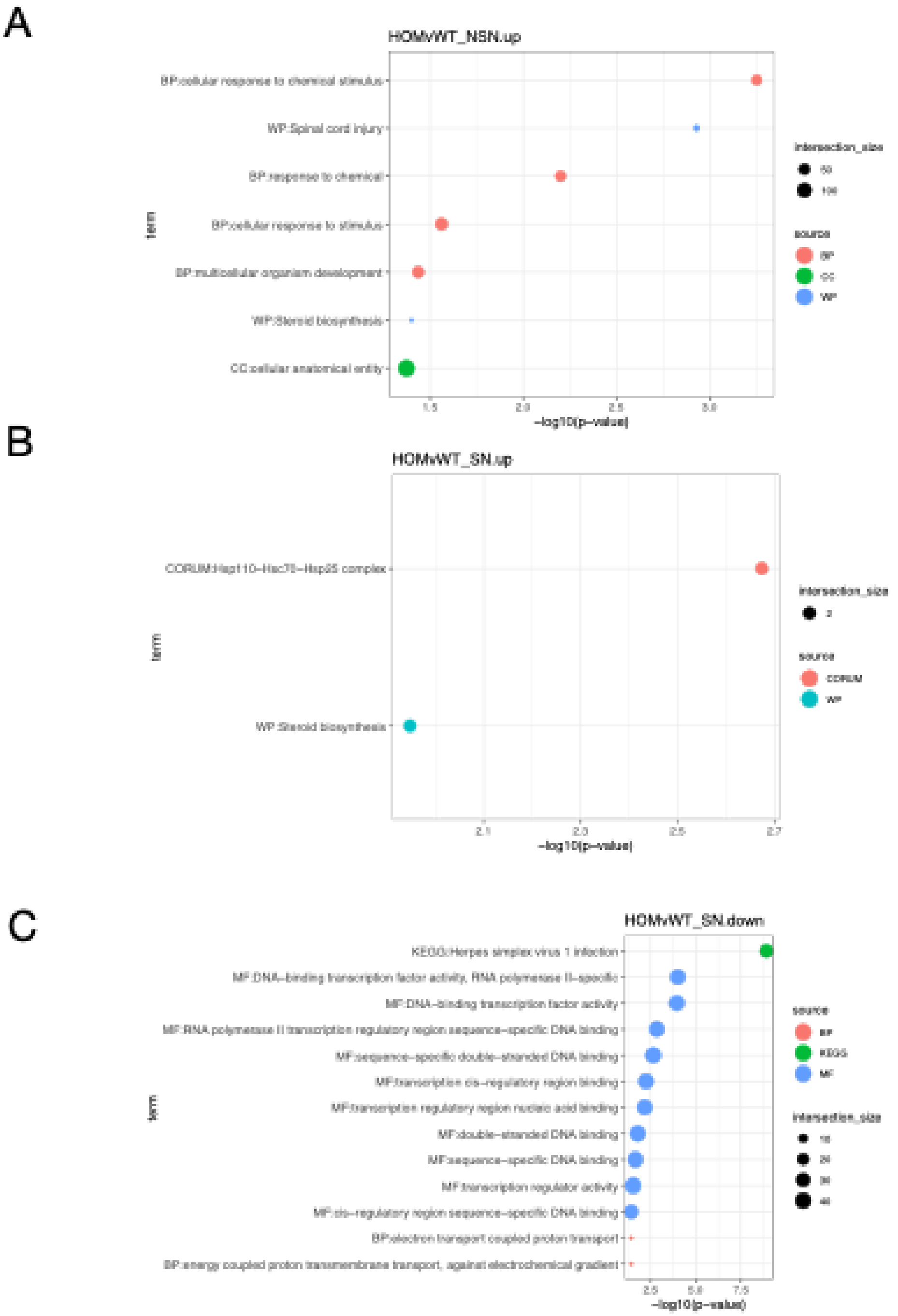

Supplementary Figure S5: Enrichment analysis of RNA-seq DEGs in Germinal Vesicle stage *Nlrp5 -/-* oocytes. 282 DEGs in the non-surrounded nucleolus (NSN) stage *Nlrp5 -/-* cohort. 347 DEGs in the surrounded nucleolus (SN) stage *Nlrp5 -/-* cohort. **(A)** Gene ontology for the 178 upregulated DEGs in NSN *Nlrp5 -/-* samples. Although 104 DEGs were downregulated in the *Nlrp5 -/-* cohort, gene ontology for these downregulated genes produced no hits. **(B)** Gene ontology for the 76 upregulated DEGs in SN *Nlrp5 -/-* samples. **(C)** Gene ontology for the 271 downregulated DEGs in SN *Nlrp5 -/-* samples. Analysis performed in R using gprofiler. Dot size denotes number of proteins intersecting with database term. Source legend lists the database for each hit by colour. BP = GO biological process, CC = GO cellular component, MF = GO Molecular function, WP = Wiki Pathways, CORUM = comprehensive resource of mammalian protein complexes, KEGG = Kyoto Encyclopaedia of Genes and Genomes. The REACTOME database also used but generated no hits.

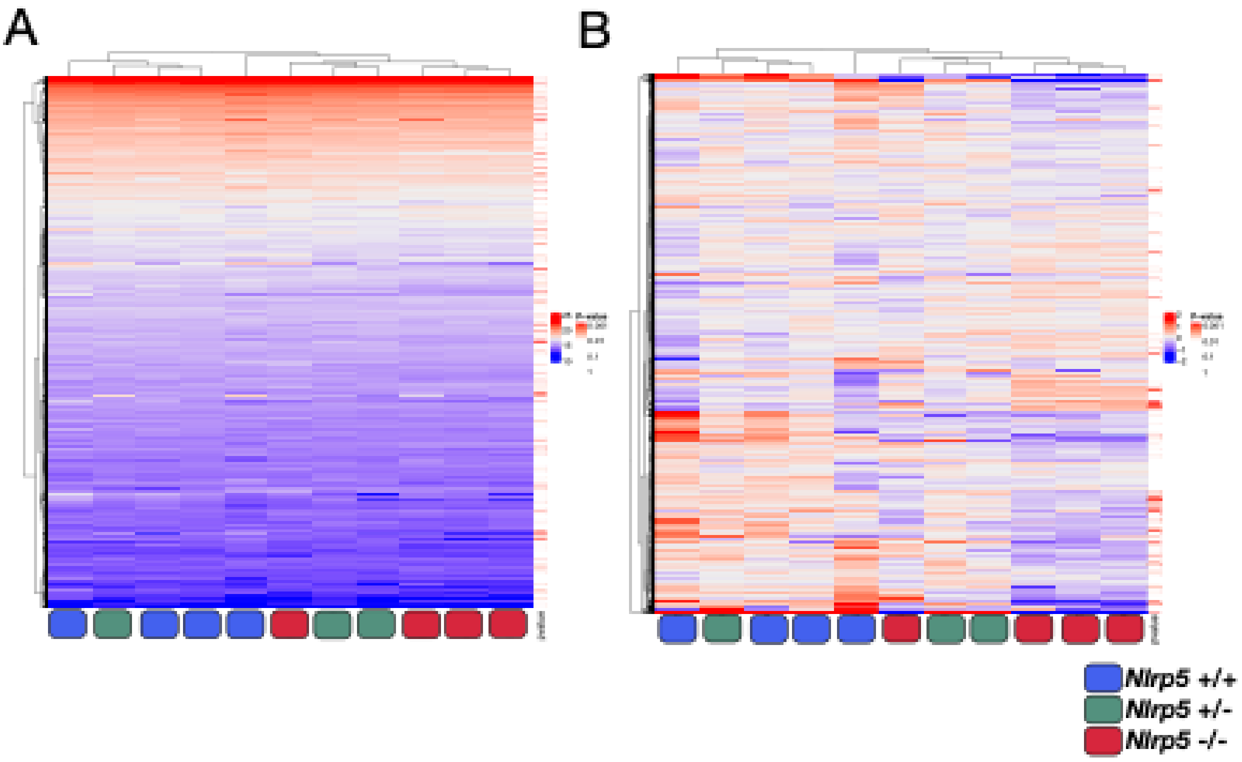

Supplementary Figure S6: Hierarchical clustering of raw protein abundance values between bulk germinal vesicle stage oocyte samples of the three genotypes. **(A)** Log_2 abundances heatmap. **(B)** Log_2 with mean subtracted. *Nlrp5 -/-* samples cluster with some *Nlrp5 +/-* samples on one side, while *Nlrp5 +/+* (WT) samples cluster with one *Nlrp5 +/-* sample at the other side. p-values in right-most column (dark red denotes p-value <=0.001).

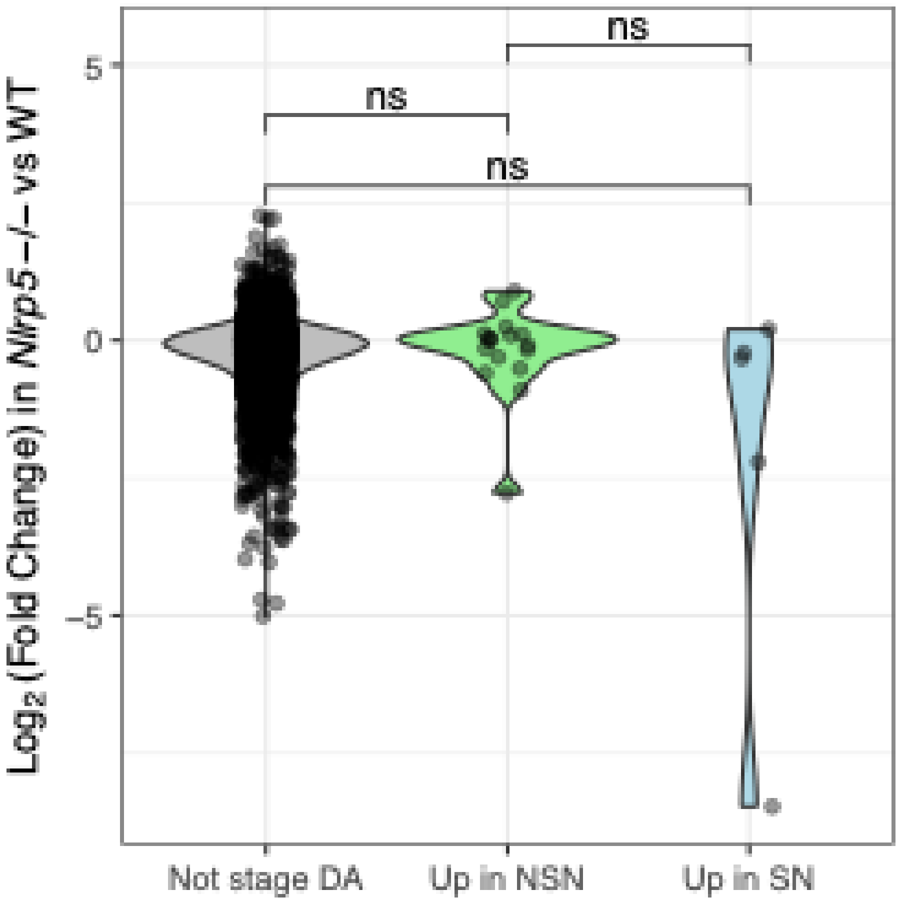

Supplementary Figure S7: Violin plot showing the $\log_2$ fold change of each protein detected in the *Nlrp5 -/-* oocyte proteomics data. Black = proteins in dataset that are not listed as differentially abundant in the Monti et al, 2013 wild type proteomic data. Green = proteins listed as 'significantly increased in abundance' in non-surrounded nucleolus (NSN) oocytes from Monti et al, 2013 study. Blue = proteins listed as 'significantly decreased in abundance' in NSN oocytes from Monti et al, 2013 study (NALP5 is included in this category, bottom right). Significance test is a t-test for significance (significant where $p < 0.05$. ns = not significant). Each point is a protein.

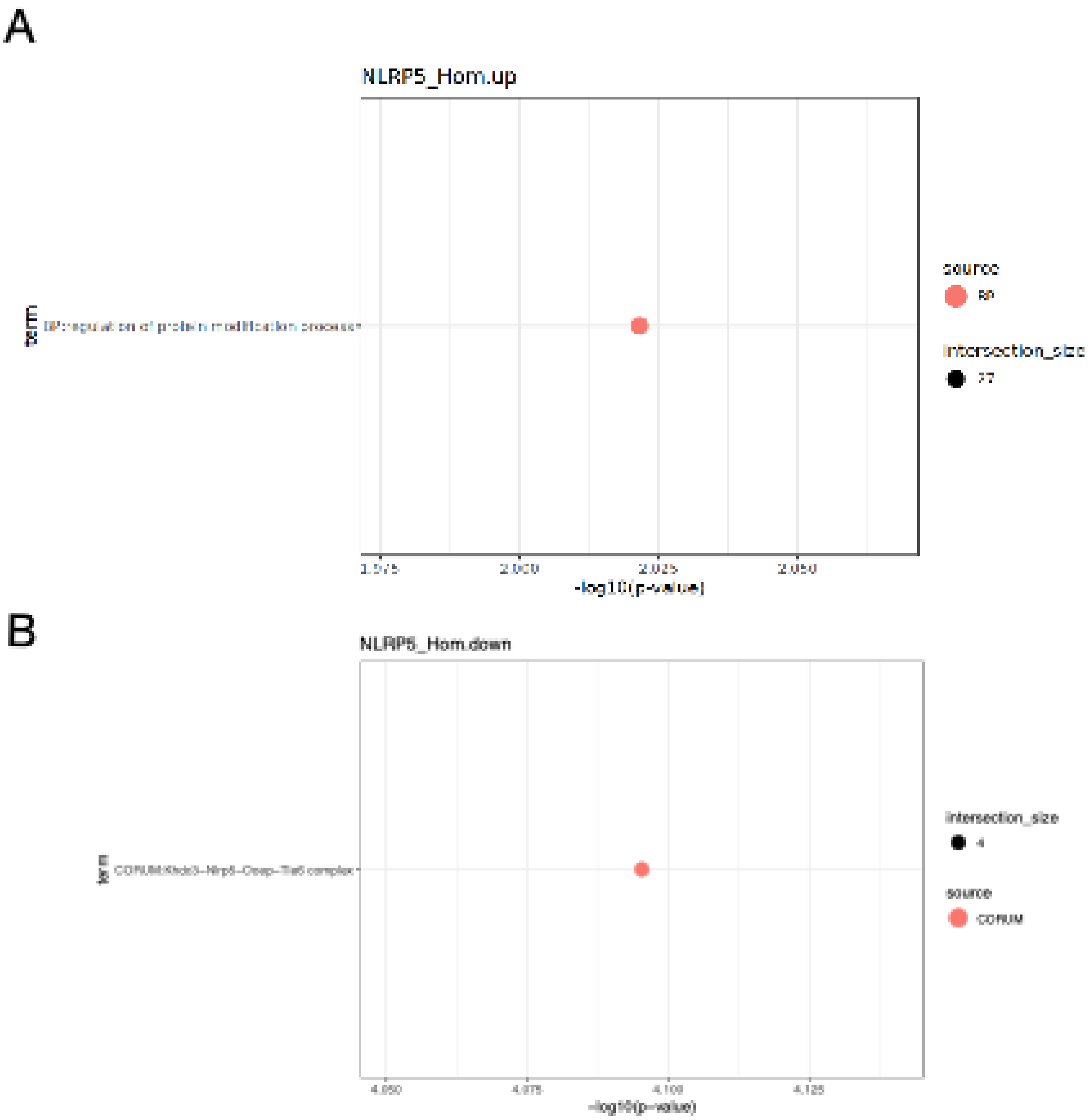

Supplementary Figure S8: Enrichment analysis of differentially abundant proteins in *Nlrp5 -/-* samples. **(A)** Upregulated proteins, **(B)** downregulated proteins. Based on 370 differentially abundant proteins (FDR < 0.1, log2 fold change of <-0.5 or > 0.5, protein abundance altered by 30% or more). Analysis performed in R using gprofiler. Dot size denotes number of proteins intersecting with GO/KEGG/Reactome database term. Source legend lists the database for each hit by colour (BP = GO biological process, CORUM = comprehensive resource of mammalian protein complexes).

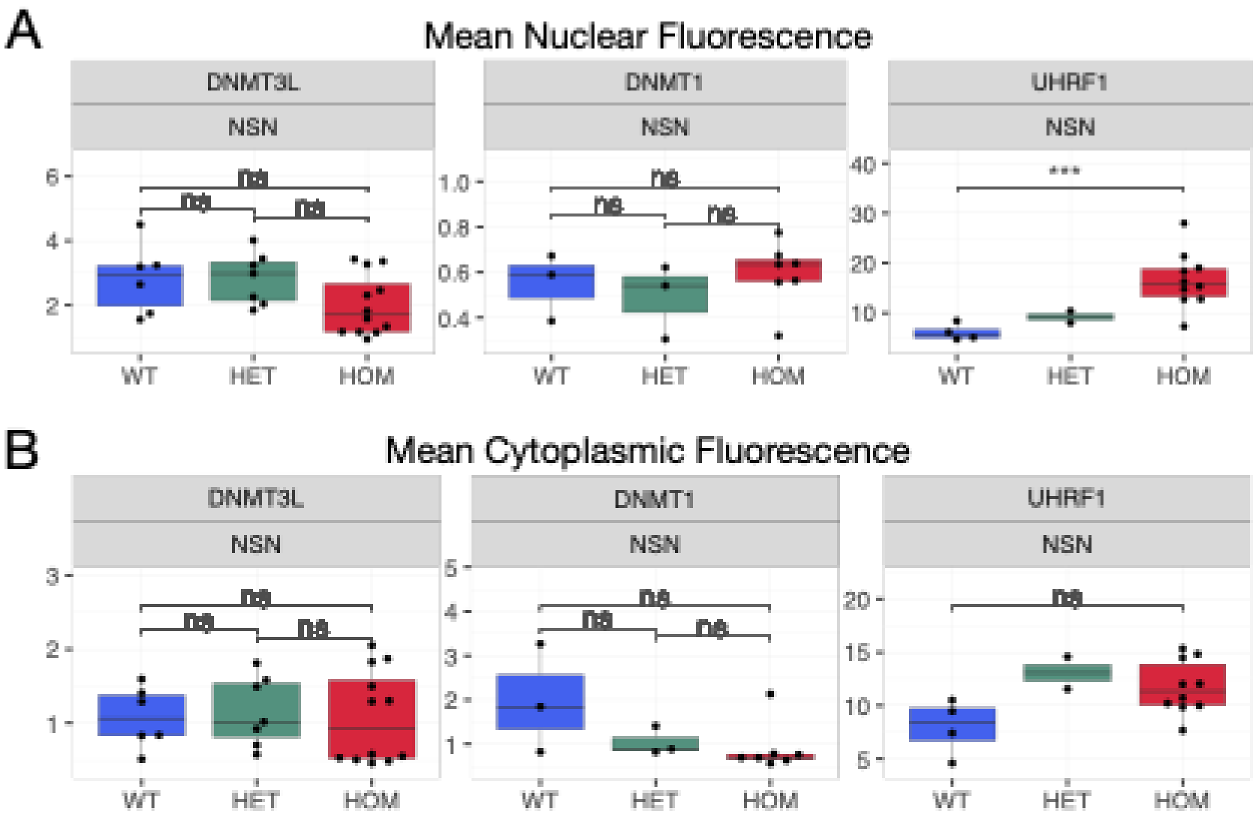

Supplementary Figure S9: Mean nuclear and cytoplasmic fluorescence values for non-surrounded nucleolus germinal vesicle stage oocytes of each genotype. (A) Relative mean nuclear and **(B)** relative mean cytoplasmic fluorescence values for non-surrounded nucleolus (NSN)-stage DNMT3L, DNMT1 and UHRF1 in germinal vesicle (GV) oocytes, normalised by γ-tubulin fluorescence. There was no difference in significance or trends when normalised by background instead. GV oocytes collected from 8-week-old mice. Shapiro-Wilk normality test applied. If $p>0.05$, students t-test was used. If $p<0.05$, Wilcoxon Rank-Sum test was used. * Significant ($p<0.05$) at 20% FDR. WT = *Nlrp5 +/+* oocytes, HET = *Nlrp5 +/-* oocytes, HOM = *Nlrp5 -/-* oocytes.

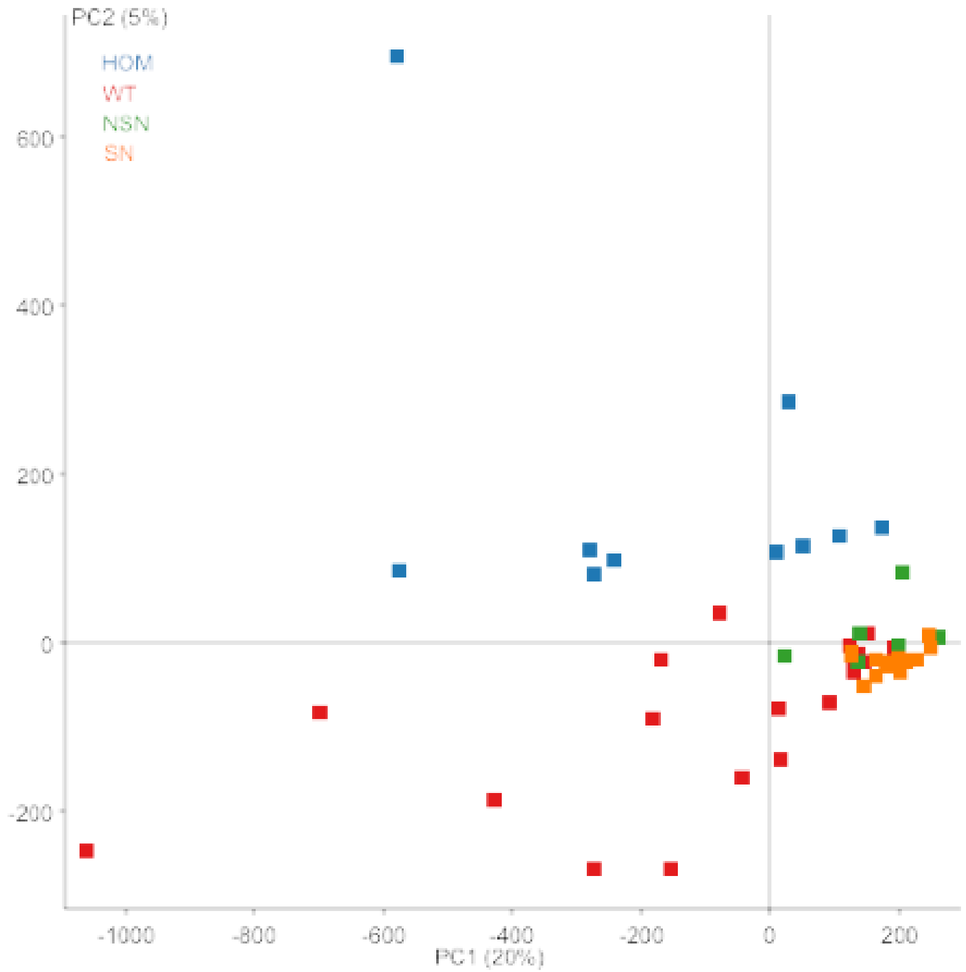

Supplementary Figure S10: ScPBAT-sequencing PCA embeddings of GV-stage oocytes from the present study and the NSN-SN staging dataset. Embedding of each *Nlrp5 -/-* (HOM, blue) and *Nlrp5 +/+* (WT, red) scPBAT-seq sample in a PCA space, along with each non-surrounded nucleolus (NSN, green) and surrounded nucleolus (SN, orange) sample from the NSN-SN staging dataset (Demond et al., 2024). Probes for plotting PCA of single oocytes were defined using bulked samples: probes were built over hypo/hyper methylated domains, which were quantified in all 4 bulk samples and overlapping genes (19703 probes). PCA was then performed on methylation scores in the single oocyte samples, using these 19703 probes.
