# Supplementary material for: An Nlrp5-null mutation leads to attenuated de novo methylation in oocytes, accompanied by a significant reduction in DNMT3L
Source: Mol Hum Reprod. 2025 Nov 18;31(4):gaaf055. doi: 10.1093/molehr/gaaf055 (PMC12704423; doi:10.1093/molehr/gaaf055)
Supplement: gaaf055_Supplementary_Data [file Review_comments_and_author_responses_MHR-25-0090.R2 _supplementary_information.pdf › Review_comments_and_author_responses_MHR-25-0090.R2 _supplementary_information.pdf]

Reviews and authors' responses for manuscript 'An *Nlrp5*-null mutation leads to attenuated *de novo* methylation in oocytes, accompanied by a significant reduction in DNMT3L' by Leah Nic Aodha et al.

Original submission: MHR-25-0090

Reviewer 1

The manuscript describes the analysis of oocytes from NLRP5 null mice with the hope to better understand NLRP5 role in the abnormal DNA methylation of multilocus imprinting defects and biparental hydatidiform moles. The authors performed mainly a transcriptomic and proteomic analyses on oocytes from null, het, and wt mice and analyzed the data along with some existing dataset from PADI6-/- and other mouse models.

Comments:

The data and the analyses are nice and the manuscript is well-written and interesting. This study revealed some earlier abnormalities in the oocytes before the germinal vesicle breakdown that have not been reported before, which is an interesting finding.

At the end of the abstract, the authors state that "This provides evidence for mechanisms leading to downstream misregulation of imprinted genes, which in turn, may result in imprinting syndromes, multi-locus imprinting disturbances (MLID) and hydatidiform moles" For me, this statement is strong and simplistic because it is not known if the MLID and hydatidiform moles phenotype are the results of the reduction in DNMT3L. The abnormal methylation is one of many abnormalities in the oocytes, depending on what people assess (or able to assess) and altogether, all the abnormalities, are responsible for the above phenotypes.

I would say, the misregulation of imprinted genes is associated with imprinting syndromes, MLID, and hydatidiform moles but whether the phenotypes are the consequence of their misregulation, has not been demonstrated yet, at least for hydatidiform moles. I would say, "…may contribute to imprinting syndromes,…….". Perhaps, the earlier abnormalities, some of which were revealed in this study, could be also contributing to the phenotypes.

Reviewer 2

The manuscript "An Nlrp5-null mutation leads to attenuated de novo methylation in oocytes, accompanied by a significant reduction in DNMT3L" presents a multi-omic analysis of a Nlrp5-null mouse model, focusing on the impact of this mutation on the subcortical maternal complex (SCMC), proteome, transcriptome, and DNA methylation in oocytes. The study provides valuable insights into the mechanisms linking SCMC to epigenetic regulation, with implications for infertility.

The manuscript is well-structured, most of the methods are described in sufficient detail and the figures generally support the findings. The discussion integrates the data and connects the findings to existing literature. There are some points where the manuscript could be strengthened for clarity.

Major Points

In Figure 1B, although the change in NALP5 subcellular localization is clear, the intensity variation in the heterozygote is not noticeable. It does not appear to be due to secondary antibody accumulation, but to confirm this, the authors need to present negative controls along with a quantification assay for this specific protein, since proteomics only showed a slight decrease.

How the authors explain the DNMT3L reduction by over 75% in -/- oocytes without any changes evidenced by immunofluorescence except by the cellular localization? In addition, while the reduction of DNMT3L protein is shown, the functional impact on global DNA methylation is modest (approximately 5% global reduction). Given this, how do the authors interpret the biological relevance of these methylation changes? Are these sufficient to cause imprinting defects?
Could the authors provide additional evidence (e.g., pattern of methylation of imprinted genes or gene expression of these genes in embryos) to directly link the observed methylation changes to downstream developmental defects?

The manuscript uses FDR < 0.1 and log2 fold change cutoffs of 0.5 for significant changes. It is necessary to justify this choice and discuss the impact on false positives.

Figure 7C shows only gDMR CpG methylation with a "borderline but not significant p value (significance cutoff <= 0.05)". However, Figure 7A demonstrates a significant reduction in global CpG methylation. Clarify the discrepancy between this data.

The authors could better explore the implications of the UHRF1 findings in the context of DNMT1. While the authors conclude UHRF1 is "unlikely to affect methylation maintenance in the Nlrp5-null due to the lack of DNMT1 effect", the altered localization of UHRF1 and its mild reduction might still have some effect or participate in a complex regulatory mechanism involving the SCMC.

The manuscript points out that DNMT3L is not expressed in human oocytes, limiting the DNMT3L finding to human imprinting disorders caused by SCMC mutations. The author should discuss the potential alternative mechanisms in humans.

The low correlation between transcript and protein abundance changes suggests post-transcriptional regulation. The authors could better explore mechanisms underlying this discordance, for example, protein stability or degradation pathways influenced by SCMC.

The analysis of scPBAT-data can be improved as the definition of “probes” is not clear as well as how the hypo/hyper-methylated domains in tiles were defined. Chi Square test seems to be not appropriated for this type of comparison. Regarding the multi-omic analysis, although the hypergeometric test is correct, the authors should also observe other parameters. For instance, in both proteomic and transcriptome data “control of gene expression” is one of the most affected pathways (even not by the same transcripts or proteins) and this was not mentioned in the manuscript.

Minor points
At times, the manuscript uses different terms for the same model (e.g., Nlrp5 -/-, Nlrp5 KO)

Include a list the NSN/SN marker genes used for classification in Table S1(i) as referenced

Some figure legends (e.g., Figures 4 and 5) could be more detailed to improve clarity.

Associate Editor

This manuscript utilised CRISPR/Cas9 technology to create a new Nlrp5 (also known as Mater) knockout mouse line, a key component of the subcortical maternal complex (SCMC), and explored the maternal roles of Nlrp5. By applying multi-omics approaches (transcriptomic, proteomic, and DNA methylome analyses), the authors reported developmental and molecular defective phenotypes. Both reviewers acknowledged the merit of this study by providing new insights into the biological role during the NSN-SN oocyte transition as well as the regulatory role of the SCMC complex linked to epigenetic regulation. However, Reviewer 2 noted concerns about the IF data for NALP5, as the images differed from previous reports (Kim et al., Dev Biol 2014). The reviewer suggested including additional controls. I also recommend that the authors provide either new data using Triton X extraction (Kim et al., PLoS One 2010) or primary antibody-conjugated IF labelling (Jentoft, Cell 2023) to support Figure 1.

Additionally, both Reviewers 1 and 2 pointed out the limitation of linking this Nlrp5 mouse model to human MLID and hydatidiform mole phenotypes due to the lack of DNMT3L expression in human oocytes.

The authors should address these primary comments, as well as Reviewer 2’s major concerns related to statistics and bioinformatics analyses concerning CpG methylation and imprinting loci.

Editor-in Chief

This is a highly rated study that was conducted to investigate the role of a novel specific mutation in Nlrp5 (a.k.a. Mater) - one of the components of the subcortical maternal complex. The Authors introduced the mutation (stop codon) in the mouse locus using CRISPR/Cas9 technology, obtaining Nlrp5 -/- null oocytes; these were examined during oogenesis and embryonic development in comparison to Nlrp5 +/- and Nlrp5 +/+ counterparts in terms of gene expression (proteome, transcriptome, DNA methylation). Oocytes were examined at the GV stage, with an interesting subgrouping into SN-like and NSN-like. Among the main conclusions, the Author reported that Nlrp5 -/- oocytes had defects of meiotic maturation, were unable to develop past the 2-cell stage, and presented a range of protein level alterations in oocytes, including epigenetic modifiers such as Dnmtl3L. The altered proteome underlies impaired oocyte developmental competence, possibly mediated by low level of global DNA hypomethylation. After peer review there is a strong consensus that the manuscript has high scientific merit and deserves publication, not in the present form however. The two Reviewers identified aspects that require attention, and the Associate Editor summarized the aspects that should be addressed in a successful revision. In particular, the immunofluorescence of Nlrp5 oocytes (Fig.1B) should be improved, the proposed link between Nlrp5 mutation and dysregulation of downstream imprinted genes should be mitigated, and aspects of the statistical and bioinformatic analysis should be justified (e.g. threshold used to make the call of differentially expressed protein, at absolute log2 fold change of 0.5 with FDR <0.1).

Our Reviewers and Associate Editor made also other remarks, which the Authors should take to heart. In addition to the comments of Reviewers and Associate Editor, the Editor in

Chief would like to note the following aspects, and kindly ask the Authors to explain. First, EiC is a little surprised that no MII oocytes were examined, although these are the actual starting material for the embryos and for the disturbance of the epigenome during embryogenesis. In addition, the cortical enrichment of the SCMC (assuming it occurs, despite the findings of Jentoft et al.) would be more pronounced in MII than in GV stage. It is not clear if the Authors used, or not, gonadotropin stimulation to collect the GV oocytes. Collecting GV oocytes from a non-stimulated ovary means that the oocytes are very heterogeneous (also in size); besides, without PMSG there won't be many antral follicles to poke/scratch. Given the time elapsed from CRISPR/Cas9 mutagenesis to phenotypic analysis (19 days to birth, plus 3-4 weeks after birth), the possibility of adaptive or compensatory effects should be taken into consideration. Some key statements, such as the reduction of this or that protein of interest (especially NLRP5 and DNMT3L) are based on saturated immunofluorescence images (Fig.1B), on nuclear to cytoplasmic ratios that may be confounded by the different sizes of the oocytes (Fig.6 A,B; Nlrp5 -/- oocytes look smaller), or on heat maps. The heat maps are especially an issue for the EiC. Example: "Nlrp5 -/- bulk oocyte samples showed a log2 fold change for NALP5 of -8.47 compared with Nlrp5 +/+ oocytes, which corresponds to 0.3% of the Nlrp5 +/+ level. Nlrp5 +/- samples had a far lower but still significant log2 fold change of -0.55, corresponding to 68% of the Nlrp5 +/+ NALP5 level." EiC is of the opinion that for the main proteins of interest the Authors should show the measured values in a main table (not supplementary) instead of heat maps based on log2 values with means subtracted (e.g. Figure 4A, 5A). Since the Authors have conducted mass spec analysis in data independent acquisition mode using biological replicates ("3-4 bulk oocyte samples per genotype (33 GV oocytes per bulk sample)"), EiC kindly asks to produce a main table (not supplementary) showing the real measured values (not log2 abundance values with mean subtracted). This main table should include e.g. NLRP5, DNMT3L, UHRF1, KDM2B and DNMT1; essentially, Figure 4A and 5A should be replaced or flanked with tables that allow the reader to see the measured values without having to do conversions. The threshold used for making the call of significant difference is, for proteomic analysis, rather low (log2 0.5 means 40% in linear scale, from 1 to 1.4, and such differences probably exist between technical replicates, let alone biological replicates). This study is deemed highly valuable by the Journal, and so we wish it to become 'perfect,' hoping that these comments from the editorial team will help in that regard. The Authors are certainly in a strong position to produce a successful revision of this manuscript. The Journal looks forward to receiving the revised version of this valuable and highly rated work, and publishing it.

Authors' Response to Decision Letter for MHR-25-0090

Comments from the Editor-in-chief and Associate Editor

We are grateful that the Editor-in-chief, Associate Editor and reviewers rated this study highly, that it has high scientific merit and is worthy of publication, and we very much appreciate all the constructive suggestions for appropriate revisions.

In relation to the EiC's comments that:

(i) the immunofluorescence of Nlrp5 oocytes (Fig.1B) should be improved – please see responses **#4, #10** below;

(ii) the proposed link between Nlrp5 mutation and dysregulation of downstream imprinted genes should be mitigated – please see response **#6** below;

(iii) aspects of the statistical and bioinformatic analysis should be justified – please see responses **#19-21**, in particular, below.

In relation to the EiC's specific comments:

1. The EiC is a little surprised that no MII oocytes were examined, although these are the actual starting material for the embryos and for the disturbance of the epigenome during embryogenesis. In addition, the cortical enrichment of the SCMC (assuming it occurs, despite the findings of Jentoft et al.) would be more pronounced in MII than in GV stage.

The EiC is correct that the MII oocyte represents the ultimate stage of the gamete prior to fertilisation and that, in addition to its own unique epigenome, it contains all the components for the epigenetic reprogramming of both parental genomes in the zygote.
We focussed on GV oocytes in our study in large part because of the ability to make comparisons with datasets from other studies that prioritised analysis of GV or fully-grown ovarian oocytes (e.g., Refs 3, 14, 23). In addition, from our own experience we found the *Nlrp5-/-* oocytes to be particularly fragile after superovulation (which we employed with IVF in order to obtain zygotes for time-lapse imaging of embryos), so that we were concerned that this would compromise some of the molecular properties we were most interested in, such as the transcriptome and proteome.

2. It is not clear if the Authors used, or not, gonadotropin stimulation to collect the GV oocytes. Collecting GV oocytes from a non-stimulated ovary means that the oocytes are very heterogeneous (also in size); besides, without PMSG there won't be many antral follicles to poke/scratch.

The EiC raises a valid question. We chose to collect GV oocytes from unstimulated mice to capture oocytes in their natural physiological state and avoid potential confounding effects of hormonal stimulation on oocyte quality and molecular profiles – certainly, we had observed that *Nlrp5-/-* MIIs obtained after the hormonal regime for superovulation were physically more fragile. Histological analysis of ovaries from females at 3-4 weeks of age, the age at which we collected GVs, did not reveal any differences in the proportions of follicle stages in the different genotypes (as previously noted for the original *Nlrp5* knock-out; Ref. 45). Collecting from females at this age will capture the first cohort of fully-grown GV oocytes, and we did not find any notable differences in the yield of oocytes between genotypes collected by this method (an average of ~75 per female). Inspection under the microscope did not indicate any obvious skew in sizes between genotypes (although we did not routinely size them with a graticule in the interests of minimising handling time and preserving molecular quality), but we did later infer from DAPI-staining in our immunofluorescence studies that there was a deficiency of SN oocytes in the *Nlrp5* knock-out, as we report in the manuscript.

We have added a statement in the Material and Methods to indicate that GVs were obtained from ovaries without gonadotropin stimulation (lines 764-765):

"*No hormonal stimulation was employed prior to oocyte collection*."

3. Given the time elapsed from CRISPR/Cas9 mutagenesis to phenotypic analysis (19 days to birth, plus 3-4 weeks after birth), the possibility of adaptive or compensatory effects should be taken into consideration.

We think the Editor-in-Chief may have misinterpreted our experimental design, but this potential misinterpretation suggests to us that we should have been more explicit in our Methods about the generation of the CRISPR/Cas9 mutation and collection of samples. The *Nlrp5* mutation was induced by CRISPR/Cas9 mutagenesis in F0 zygotes, a male F0 that was identified to have sustained a disrupting deletion on both *Nlrp5* alleles was mated to WT (C57BL/6Babr) females to produce F1 offspring, and F1 heterozygous males and females crossed to generate the first homozygous F2 females, in which oocytes were first recovered to confirm ablation of the NLRP5 protein by IF. Most oocytes for the various analyses were collected from homozygote females (and littermate-matched heterozygote controls) obtained from F2 and later intercross generations, to allow for expansion of the breeding stock of the KO strain. Therefore, there are multiple generations between induction of the mutation in zygotes by CRISPR/Cas9 and the collection of oocytes from homozygous females. Although there is a potential for off-target effects in any CRISPR/Cas9 mutagenesis, despite optimal sgRNA design, off-target effects will have been segregated away in these subsequent generations (unless in very close linkage to the *Nlrp5* locus). In addition, unless there is a haploinsufficiency phenotype for the *Nlrp5* mutant allele (for which we have no evidence from the breeding data; heterozygous females produced litters in matings with WT males with no statistical reduction in offspring number), there is no obvious scope for compensatory effects, as homozygous null females are not bred from as they are infertile. To clarify our experimental design, we have revised the Methods section, lines 743-744, by adding the statement:

"*Homozygous and heterozygous mutant mice for collection of oocytes were obtained at the F2 and subsequent generations*"

4. Some key statements, such as the reduction of this or that protein of interest (especially NLRP5 and DNMT3L) are based on saturated immunofluorescence images (Fig.1B), on nuclear to cytoplasmic ratios that may be confounded by the different sizes of the oocytes (Fig.6 A,B; Nlrp5 -/- oocytes look smaller).

As noted in response **#10** below, we are not using immunofluorescence analysis to achieve precise estimates of protein levels, which are far better obtained from the highly quantitative mass-spec analysis, but use the immunofluorescence as an orthogonal confirmation of profound changes (as in the case of NALP5), or to explore possible effects on protein localisation (for DNMT1, UHRF1, DNMT3L). Relating in particular to the saturated images for NALP5 (**Figure 1B**), the over-exposure was done to demonstrate the absence of signal in the *Nlrp5-/-* oocytes. As noted in response **#10** below, we have added

immunofluorescence on Triton-X extracted oocytes to get a better view of the distribution of NALP5 protein in wild-type and *Nlrp5+/-* GVs and to confirm absence in *Nlrp5-/-* GVs (**new Figure 1C**). Owing to the extreme fragility of oocytes after Trtion-X extraction, we were unable to obtain sufficient datapoints for a reliable quantitative comparison of fluorescence between *Nlrp5+/+* and *Nlrp5+/-* GVs; therefore, we have removed the statements from the text on reduced NALP5 abundance in *Nlrp5+/-* GVs that were based on immunofluorescence imaging.

5. EiC is of the opinion that for the main proteins of interest the Authors should show the measured values in a main table (not supplementary) instead of heat maps based on log2 values with means subtracted (e.g. Figure 4A, 5A). Since the Authors have conducted mass spec analysis in data independent acquisition mode using biological replicates ("3-4 bulk oocyte samples per genotype (33 GV oocytes per bulk sample)"), EiC kindly asks to produce a main table (not supplementary) showing the real measured values (not log2 abundance values with mean subtracted). This main table should include e.g. NLRP5, DNMT3L, UHRF1, KDM2B and DNMT1; essentially, Figure 4A and 5A should be replaced or flanked with tables that allow the reader to see the measured values without having to do conversions.

We would prefer to retain the heatmaps of these selected proteins in **Figures 4A, 5A** because they provide a presentation of the data that gives an immediate impression of changes in protein abundance, which is useful to readers. But we also appreciate the value of providing the raw measured protein abundance values to enhance transparency and facilitate data interpretation. Therefore, we have generated two new main tables (**Tables 1 & 2**) including the raw abundance values for key proteins of interest – NLRP5, DNMT3L, UHRF1, KDM2B, DNMT1, etc., – derived from the data independent acquisition mass spectrometry analysis with biological replicates as described. We tried incorporating these tables as panels to flank the heatmaps in these figures, but the number of columns required to present all biological replicates made the numerical values too small to discern.
As before, the raw counts and log2-transformed counts for our full proteomics dataset are provided in **Supplementary Tables S2(i)**.

5. The threshold used for making the call of significant difference is, for proteomic analysis, rather low (log2 0.5 means 40% in linear scale, from 1 to 1.4, and such differences probably exist between technical replicates, let alone biological replicates).

We explored the proteomic mass-spec data within the Perseus software using a variety of FDR and log2 fold-change cut-offs. The cut-offs ultimately selected are perhaps lenient and will inevitably lead to a proportion of false positives, but we chose them to output a set of proteins, for example, to be sufficient for a meaningful gene ontogeny/enrichment analysis. Importantly, we provide the FDR, fold-change, and p-values for the list of differentially abundant proteins in **Supplementary Table S2(ii)**, which can be sorted for difference (log2 FC), log p-value or FDR by readers if they wished to focus on proteins with the greatest or the statistically most robust differences. As noted above, we also provide the raw values for all proteins in our mass-spec data (**Table S2(i)**) to enable a full exploration of the dataset

Reviewers' specific comments

Reviewer 1:

6. At the end of the abstract, the authors state that "This provides evidence for mechanisms leading to downstream misregulation of imprinted genes, which in turn, may result in imprinting syndromes, multi-locus imprinting disturbances (MLID) and hydatidiform moles". For me, this statement is strong and simplistic because it is not known if the MLID and hydatidiform moles phenotype are the results of the reduction in DNMT3L. The abnormal methylation is one of many abnormalities in the oocytes, depending on what people assess (or able to assess) and altogether, all the abnormalities, are responsible for the above phenotypes. I would say, the misregulation of imprinted genes is associated with imprinting syndromes, MLID, and hydatidiform moles but whether the phenotypes are the consequence of their misregulation, has not been demonstrated yet, at least for hydatidiform moles. I would say, "…may contribute to imprinting syndromes,…….". Perhaps, the earlier abnormalities, some of which were revealed in this study, could be also contributing to the phenotypes.

We agree that the original phrasing could overstate our findings. For example, as we remark in our Discussion, and in response #17 below, the significance of our observed effect on DNMT3L levels for human pathology is unclear, so we are deleting the statement in the abstract relating to MLIDs and hydatidiform moles. We have thus amended the closing sentences of the Abstract to (lines 44-46) as a more accurate reflection of the scope of our results.

"*Amongst impacted proteins are several epigenetic modifiers, including a substantial reduction in DNMT3L; we show that de novo DNA methylation is attenuated in Nlrp5-null oocytes, including at some imprinting control regions. This provides evidence for a mechanism of epigenetic impairment in oocytes which could contribute to downstream misregulation of imprinted genes.*"

Reviewer 2:

7. At times, the manuscript uses different terms for the same model (e.g., Nlrp5 -/-, Nlrp5 KO).

This has been rectified in the revised manuscript. We now use the term *Nlrp5-/-* throughout in the text, but for some figure labelling retain the terms WT, HET, HOM for clarity, especially when all biological replicate samples are listed.

8. Include a list the NSN/SN marker genes used for classification in Table S1(i) as referenced.

These marker genes are listed in **Supplementary Table S(i)**, which we have reformatted in the updated file for clarity.

9. Some figure legends (e.g., Figures 4 and 5) could be more detailed to improve clarity.

Additional detail has been added to the legends for **Figures 4** and **5** as requested. We have also taken this opportunity to provide additional information in some other figure legends for completeness and greater context for readers. For example, we have added the numbers of features (100-CpG tiles, gene bodies, CpG islands, etc.) analysed in the various panels in **Figure 7**.

10. In Figure 1B, although the change in NALP5 subcellular localization is clear, the intensity variation in the heterozygote is not noticeable. It does not appear to be due to secondary antibody accumulation, but to confirm this, the authors need to present negative controls along with a quantification assay for this specific protein, since proteomics only showed a slight decrease.

We agree that the IFs for NALP5 in **Figure 1B** do not adequately demonstrate changes in protein level in the heterozygote GVs; this is likely because the images are over-saturated because we wished to use the same imaging settings to show convincingly absence of NALP5 in the *Nlrp5-/-* oocytes, as well as the apparent difference in antibody or secondary antibody penetration leading to altered distribution of the fluorescence signal in the *Nlrp5+/-* oocytes. We have repeated the IFs for NALP5 localisation using Triton-X extracted GV oocytes, which has been reported to overcome some of the penetration problems for staining SCMC/CPL components (e.g. Ref. 32). This reveals a uniform cytoplasmic distribution of NALP5 IF signal (in new **Figure 1C**), as previously reported (e.g., Ref. 32). However, as noted in response **#4** above, we were unable to obtain data from sufficient *Nlrp5+/+* oocytes in this experiment for reliable comparative quantification of the IF signals owing to the fragility of the GVs after Triton-X extraction. Therefore, we are removing some of the statements regarding reduced amount of NALP5 in *Nlrp5+/-* oocytes inferred from immunofluorescence (the updated text is presented below), and we hope that providing the raw abundance values for the mass-spec data for NALP5 in the new **Table 1**, as recommended by the EiC (response **#5**), gives greater clarity of a reduction in NALP5 protein in *Nlrp5+/-* compared with *Nlrp5+/+* oocytes. The new text for the immunofluorescence analysis (lines 139-145) now reads:

"*Immunofluorescence of fixed GV oocytes demonstrated that NALP5 protein is ablated in Nlrp5 -/- oocytes, and in Nlrp5 +/- oocytes the concentrated subcortical signal for NALP5 observed in Nlrp5 +/+ oocytes is diminished and becomes more evenly distributed throughout the cytoplasm (Figure 1B). Immunofluorescence analysis of Triton-X extracted GV oocytes showed a uniform signal throughout the cytoplasm for NALP5 in both Nlrp5 +/+ and Nlrp5 +/- oocytes, and undetectable signal in Nlrp5 -/- oocytes (Figure 1C).*"

The **legend** to **Figure 1** (lines 153-156) has been updated with the explanation for the new panel 1C.

We also decided to delete the concluding statement after line 149.

“The fact that NALP5 is one of the most abundant proteins in the oocyte and a significant reduction in NALP5 in Nlrp5 +/- oocytes (as revealed by proteomics, below) reduces this subcortical saturation and leads to a more evenly dispersed cytoplasmic staining supports recent studies that indicate presence of the SCMC throughout the oocyte cytoplasm[3].”

because it becomes unnecessary after the addition of the Triton-X extracted immunofluorescence images.

We have also updated the Methods section to include the method used for the Trtion-X extraction (lines 802-806).

“For Triton extraction [32], unfixed oocytes were incubated in extraction buffer containing 0.1M KCl, 20 mM MgCl2, 3 mM EGTA, 20 mM HEPES (pH 6.8), 0.1% Triton X-100 and 1× Complete Protease Inhibitor Cocktail (Roche) for 10 minutes and rinsed in PBS quickly and fixed and permeabilised as above.”

And added the details about the H3 antibody (lines 812-813) and the confocal microscope used (line 823).

11. How do the authors explain the DNMT3L reduction by over 75% in -/- oocytes without any changes evidenced by immunofluorescence except by the cellular localization?

I think we would recognise the relative imprecision of quantification of immunofluorescence signal depending of the quality of antibodies, the degree of background signal, cross-reactivity, the contribution of autofluorescence, etc. Therefore, any change in protein abundance inferred from immunofluorescence is likely to represent an underestimate of actual changes, unless all conditions above are perfect. The intention of the immunofluorescence was very much more to investigate the possibility of changes in protein localisation, given the reported effects of SCMC mutants on localisation of epigenetic modifiers, such as DNMT1.

12. While the reduction of DNMT3L protein is shown, the functional impact on global DNA methylation is modest (approximately 5% global reduction). Given this, how do the authors interpret the biological relevance of these methylation changes? Are these sufficient to cause imprinting defects?

Despite a ~75% reduction in DNMT3L protein detected by mass-spectrometry, we detected only a ~5% reduction in genomic methylation by scPBAT, as the reviewer points out. DNMT3L is essential for *de novo* methylation in mouse oocytes, but there is no evidence of haploinsufficiency in the literature, indicating that *de novo* methylation can be fully accomplished with a notional reduction of 50% in DNMT3L protein levels (although this has not been experimentally shown to our knowledge). As we mentioned in the Discussion (lines 615-621), we interpret our observation to suggest that *de novo* methylation is attenuated in *Nlrp5-/-* oocytes, with the expectation that there may be some genomic sites most susceptible. In this respect, it is useful to note that gDMRs do not gain methylation at the same rate during oocyte growth (PMID: 28507606); and are not all equally affected by loss of other epigenetic factors, such as KDM1B (Ref. 72 & PMID: 19727073) – unfortunately,

however, we did not find an easy overlap between such effects and the gDMRs we detected here to be most affected.

In terms of the potential biological relevance of the methylation changes we observed, it is highly unlikely that a global reduction in methylation of ~5% would have an impact on embryo and offspring development. On the other hand, reduced methylation in oocytes of a gDMR such as at *Grb10* (overall reduction of 56%; **Table S3(ii)**) could have an impact in the embryo, in particular if critical CpGs within the gDMR were undermethylated, such as within binding motifs for ZFP57 and/or ZFP445, proteins required in the early embryo to ensure maintenance of gDMR methylation (Ref. 19). Imprinted expression of *Grb10* is required for normal growth regulation pre- and post-natally (e.g., PMID: 19174477; PMID: 19833122). Loss of, or reduced, methylation of a subset of gDMRs in oocytes can significantly compromise embryo viability (e.g., PMID: 19727073). As noted in response to the reviewer's next question (**#13**), the inability to obtain embryos from *Nlrp5-/-* oocytes precludes testing this.

In light of the reviewer's question, we have added the following statement in the Discussion (lines 666-672), which should provide some context to readers:

"*It seems unlikely that the ~5% reduction in global methylation in Nlrp5-/- oocytes we observe will have an effect on early embryo development, especially given the genome-wide methylation reprogramming in the zygote. But a focal reduction in methylation of a gDMR could be more meaningful, if it involved a gDMR for an imprinted gene of developmental significance like Grb10, and occurred over features of the gDMR such as ZFP57/ZNF445 binding sites*[19] *at which methylation is critical for maintenance of imprinting and appropriate expression of the associated gene in the embryo*".

13. Could the authors provide additional evidence (e.g., pattern of methylation of imprinted genes or gene expression of these genes in embryos) to directly link the observed methylation changes to downstream developmental defects?

Unfortunately, we are unable to do this because of the inability to obtain embryos from *Nlrp5-/-* oocytes, because of the complete 1-2 cell arrest we observed.

14. The manuscript uses FDR < 0.1 and log2 fold change cutoffs of 0.5 for significant changes. It is necessary to justify this choice and discuss the impact on false positives.
– please see response **#5** above

15. Figure 7C shows only gDMR CpG methylation with a "borderline but not significant p value (significance cutoff <= 0.05)". However, Figure 7A demonstrates a significant reduction in global CpG methylation. Clarify the discrepancy between this data.

We applied different statistical tests in **Figure 7A** and **C** owing to the nature of the data: a Welch's t-test in 7A because the data are normally distributed; a non-parametric Wilcoxon rank-sum test/Mann Whitney test for 7C because the data are not normally distributed.

We think it is also useful to consider the nature of the data in these two presentations/analyses. **Figure 7A** shows the estimated global CpG methylation percentage per oocyte based on mean % methylation over all informative 100-CpG tiles in each oocyte; which indicates significant hypomethylation in *Nlrp5-/-* oocytes. As evident from **Figure 7B**, such a global estimate imperfectly represents the actual methylation status, because the mouse oocyte methylome is so bimodal, with 100-CpG tiles either overwhelmingly hypomethylated (<20%) or hypermethylated (>80%), with a minority of tiles with intermediate methylation. A generalised hypomethylation in *Nlrp5-/-* oocytes could represent dispersed and random reductions throughout the genome; for example, **Figure 7B(ii)** highlights 2452 100-CpG tiles of 152,071 in that analysis that statistically were called differentially methylated, i.e.,1.6% of the total. These tiles are called differentially methylated if there is some consistency in the effect. There will be some false discovery in this number owing to relative sparsity of coverage and sampling effects, but the strong excess of reduced methylation amongst differentially methylated tiles (78% of the 2452 tiles) is consistent with the relative hypomethylation shown in **Figure 7A**.
In **Figure 7C**, the analysis is at the level of individual whole gDMRs in data merged by genotype: these gDMRs are expected to be fully methylated in wild-type oocytes.

We have updated the legend to **Figure 7** to include information about how the global methylation percentage per oocyte was computed, and the numbers of features observed in each graph, etc.

16. The authors could better explore the implications of the UHRF1 findings in the context of DNMT1. While the authors conclude UHRF1 is "unlikely to affect methylation maintenance in the Nlrp5-null due to the lack of DNMT1 effect", the altered localization of UHRF1 and its mild reduction might still have some effect or participate in a complex regulatory mechanism involving the SCMC.

We agree that the link with UHRF1 is worth exploring: the altered localization (observed in some oocytes) and mild overall reduction of UHRF1 detected by mass-specrometry could contribute to subtle or context-dependent effects on DNA methylation. UHRF1 acts as a critical co-factor for DNMT1 by recognizing hemi-methylated DNA and specific histone marks, recruiting and allosterically activating DNMT1 at replication foci. Therefore, changes in UHRF1 localization could impair its ability to efficiently target DNMT1 to chromatin or modulate DNMT1 enzymatic activity, potentially affecting maintenance methylation fidelity. Furthermore, UHRF1 has additional roles beyond DNMT1 recruitment, including ubiquitination of histones to establish a chromatin environment permissive to maintenance methylation and participation in other regulatory complexes. This suggests that its mislocalisation might impact broader epigenetic regulation pathways that intersect with the SCMC functions. *Uhrf1-/-* GV oocytes are reported to have a reduction in global methylation of 7.9% (Ref. 14), which is greater than that observed in *Dnmt1-/-* oocytes (2.1%; Ref. 21), consistent with DNA methylation modulating roles of UHRF1 in the oocyte beyond its interaction with DNMT1. This reduction is of a similar magnitude to what we observe in *Nlrp5-/-* GVs (~5%). We did test for an overlap between regions hypomethylated in *Nlrp5-/-* oocytes and *Uhrf1-/-* oocytes (using the PBAT data from Ref. 14): ~15% of loci hypomethylated in one genotype are also hypomethylated in the other. But note that the comparison is imperfect because of differences in the datasets, comparing relatively sparse,

merged single-cell data (our *Nlrp5* KO) with far more deeply sequenced bulk PBAT samples of ~1000 GVs (so we are reluctant to include this analysis). On the other hand, *Uhrf1+/-* heterozygous oocytes might be a more appropriate comparison rather than the complete null, given that we detected a ~30% reduction in UHRF1 in *Nlrp5-/-* oocytes by proteomics – but there is no report in the literature on whether DNA methylation is reduced in *Uhrf1* heterozygous GVs. It is possible that the reduction in total UHRF1 could contribute to reduced global DNA methylation in *Nlrp5-/-* oocytes, but the mislocalisation of UHRF1 to the nucleus we see in some oocytes might tend to work in the other direction and promote aberrant methylation. Overall, we consider that the more severe reduction in DNMT3L levels is likely to be a greater contributor, and anticipate that an impact on UHRF1 stability or localisation is more likely to have consequences on methylation maintenance in the embryo.

As the reviewer notes, the observed effect on UHRF1 could have other impacts on the complex regulatory mechanism involving the SCMC. In a series of transfection experiments (in HEK293 cells), Unoki *et al*., 2024 identified that NLRP5 stabilizes UHRF1 protein in the cytoplasm, while in murine *Nlrp5-/-* fully-grown oocytes (FGOs), UHRF1 is destabilised in both the cytoplasm and the nucleus (Ref. 65), which partially overlaps our findings in *Nlrp5-/-* GV oocytes. They posit that the resulting reduction of UHRF1 in the nucleus, in either human and murine oocytes, could cause a defect in maintenance of methylation of CpGs in imprinting control regions during preimplantation embryonic development, via impaired DNMT1 targeting. We have expanded the Discussion (see below) to reflect that although DNMT1 appears unaffected, altered UHRF1 could play a biologically relevant role in methylation maintenance or regulation through complex mechanisms involving chromatin modifications and SCMC interactions, particularly post fertilisation. Further detailed mechanistic studies will be needed to dissect these potential contributions, but the failure of embryo progression from *Nlrp5*-/- oocytes would make it difficult to test this in this model.

Text added (lines 631–636):

"*While UHRF1 was also mildly reduced (~30%) in Nlrp5 -/- GV oocytes, it is not known to be haploinsufficient and thus, taken together with there being no significant effect on DNMT1 abundance or localisation in the Nlrp5 -/- GV oocytes, we think it is unlikely to account for the hypomethylation we observe. But given the interactions of UHRF1 with components of either form of the SCMC [23 65], the reduction could lead to a complexity of impairments in the oocyte, with a particular impact post-fertilisation [14].*"

We have also added that UHRF1 may have a contribution to *de novo* methylation in oocytes independent of DNMT1 (lines 622-623):

"While UHRF1 and DNMT1 can contribute to de novo methylation in oocytes[14 21], <u>and UHRF1 may have roles independent of UHRF1[14]</u>, they are particularly crucial for maintenance of DNA methylation."

We have removed the following statement (at line 631) in this section of the Discussion, because it is too speculative.

"*However, the subtlety of the hypermethylation in Padi6-null oocytes suggests that a similar phenotype in Nlrp14-/- oocytes is still plausible.*"

17. The manuscript points out that DNMT3L is not expressed in human oocytes, limiting the DNMT3L finding to human imprinting disorders caused by SCMC mutations. The author should discuss the potential alternative mechanisms in humans.

As the reviewer notes, we had briefly discussed the limitations of extrapolating our unexpected finding of reduced DNMT3L to explain the reported global hypomethylation observed in human KHDC3L-null oocytes or to human imprinted defects caused by SCMC mutations. A difficulty in discussing alternative possibilities is that the mechanism of *de novo* methylation in human oocytes is unknown. The lack of DNMT3L in human oocytes poses a problem, and although it is possible to speculate that non-catalytic isoforms of DNMT3B which exist (e.g., PMID: 33004415) could substitute for DNMT3L and heterodimerise with DNMT3A in a *de novo* methylation complex, there is clearly no functional evidence in human oocytes to take this notion beyond speculation. We have updated the Discussion (lines 678-684) to reflect this gap in knowledge and offer some alternatives:

"*While DNMT3L acts as an essential cofactor for DNMT3A-mediated de novo methylation in mouse oocytes, alternative molecular mechanisms must compensate for the absence of DNMT3L during oogenesis in humans* [74]. *The identity of these mechanisms is currently not understood but may involve other DNA methyltransferase paralogs, regulatory factors, or chromatin environment features that can establish methylation imprints independently of DNMT3L.*"

and have updated the closing sentence (lines 686-688):

"*the varied and species-specific effect of each individual SCMC-subunit*[35]*, as well as differences in the repertoire of epigenetic modifiers, means that specific human phenotypes will have to be further explored in models more similar to the human.*"

18. The low correlation between transcript and protein abundance changes suggests post-transcriptional regulation. The authors could better explore mechanisms underlying this discordance, for example, protein stability or degradation pathways influenced by SCMC.

We did consider this possibility and performed pathway and enrichment analyses for proteins and genes involved in post-transcriptional regulation, protein stability, and degradation pathways. Although our analyses did not reveal significant enrichment of these pathways, we can find examples of changed proteins that are involved in protein transport or stress responses (heat-shock, unfolded protein response: e.g., ERGIC1, HSP90A, DNAJA1, DNAJB1; **Table S2(ii)**; lines 376-379). It is worth noting that the low correlation between transcript and protein abundance changes has also been observed in other studies relating to the SCMC, such as in the *Tle6*, *Padi6* and *Uhrf1* knock-outs (e.g., Ref. 3, 23), and Jentoft et al. characterise the SCMC/cytoplasmic lattices as a compartment for stabilisation/storage of proteins in the oocyte, so we don't think our results are uncovering new concepts here.

19. The analysis of scPBAT-data can be improved as the definition of “probes” is not clear.

We apologise for the lack of information. In the analysis package we used, SeqMonk, probes are defined as discrete genomic regions over which quantitative analysis (such as methylation percentage) is performed. For DNA methylation analysis in SeqMonk, one has flexibility in how to define probes based on the biological question, data resolution, and coverage. In our analysis, we initially used 100-CpG tiled probes, as we felt that this would give the most unbiased assessment of the overall methylation levels across the genome. In this approach, the genome is divided into non-overlapping 100-CpG tiles. For quantitation within Seqmonk, each tile becomes a probe, and methylation calls at CpGs within that region are aggregated and quantified. For clarity, we have harmonised the description of these probes throughout the manuscript, and now instead of variably using ‘tiles’, ‘probes’ and ‘windows’, we simply say ‘100-CpG tiles’, and have updated the Methods section (lines 966-982), which we hope has improved clarity.

“*Analysis of the global DNA methylation was performed in SeqMonk, using initial quantification of probes corresponding to contiguous 100-CpG tiles across the genome, with a minimum of 30 calls per tile required for inclusion in analysis, resulting in 231,959 100-CpG tiles included. The 100-CpG tiles were subsequently filtered for analysis of different genomic features: either contained within hypo/hyper-methylated domains (152,071 probes; hypomethylated domains are consecutive tiles merged with methylation of 0-25%; hypermethylated domains merged tiles of 75-100%, as detailed in Ref. 62), or overlapping with hypermethylated CpG islands (> 75% methylation at least in one group, WT or Nlrp5 -/-; 1796 probes). Identification of differentially methylated regions (2452 hits, overlapping with 1402 genes) amongst the 100-CpG tiles contained within hypo/hypermethylated domains was carried out using a Chi Square test with significance cutoffs of the adjusted P-value <0.05 and % methylation difference of >25%. The differential methylation of gDMRs and their heatmap were analysed across whole gDMRs (with quantification of minimum 20 calls per gDMR). The gDMRs were identified with Chi-square test with the above cutoffs on the significance. gDMRs were only considered significantly differentially methylated if also highly covered (>100 CpG calls/1000bp; Tables S3(ii)):*”

20. The analysis of scPBAT-data can be improved [by detailing] how the hypo/hyper-methylated domains in tiles were defined.

The hypo/hyper-methylated domains are a well-established feature of the mouse oocyte DNA methylome (e.g., Ref. 62), comprising regions of coherent high-levels of DNA methylation (mostly expressed gene bodies) and low-levels of DNA methylation (non-expressed genes and intergenic regions; these have also been referred to as ‘partially-methylated domains, PMDs (PMID: 27626382)). We previously systematically assigned hypo- and hyper-methylation domains in the mouse oocyte (Ref. 62), and we have used the annotation created in that analysis in the current study. Hypomethylated domains were essentially assigned by merging consecutive 50-CpG tiles if they had methylation of 0-25%; hypermethylated domains assigned from consecutive tiles if methylation was 75-100%. We have updated the Methods section (lines 971-973) to include this information, although it can be found detailed in Ref. 62.

"*hypomethylated domains are consecutive tiles merged with methylation of 0-25%; hypermethylated domains merged tiles of 75-100%, as detailed in Ref. 62.*"

21. The analysis of scPBAT-data can be improved as a Chi Square test seems to be not appropriated for this type of comparison.

We understand the reviewer's point, but the decision to use a Chi squared test was determined by the need to merge (pseudobulk) the scPBAT datasets to obtain sufficient coverage of features. scPBAT yields untargeted and partial coverage of the genome; in the current study, the coverage in each oocyte is on overage ~1.9 million of the ~22 million CpGs in the mouse genome. In previous studies using scPBAT, we have randomly merged subgroups of oocytes and performed differential methylation analysis between multiple combinations of random subgroups (e.g., Ref. 63); each subgroup has more complete (but incomplete) coverage and the subgroups enable some degree of technical variation to be taken into account. However, in that study we had on average at least twice the coverage per oocyte as well as at least twice the number of samples to make that a viable approach. With the reduced depth in the current study, we have single pseudobulked groups. Although there will be an element of false discovery in any set of differentially methylated features we call, we can mitigate this by enforcing a minimum number of CpG calls per 100-CpG tile for inclusion, and then increasing the informative observations per feature by merging tiles into larger analytical units (gene bodies, CpG islands, etc.) at the same time reducing the number of features tested (and hence reducing the burden of multiple testing).

22. Regarding the multi-omic analysis, although the hypergeometric test is correct, the authors should also observe other parameters. For instance, in both proteomic and transcriptome data "control of gene expression" is one of the most affected pathways (even not by the same transcripts or proteins) and this was not mentioned in the manuscript.

We are unclear of the reviewer's meaning here, because "control of gene expression" is not a significant pathway in either RNA-seq or proteomic enrichment analysis (at least using our parameters). Even with the relatively lenient cut-off we applied for detecting differentially abundant proteins, enrichment analysis yielded only single terms for up- and down-regulated proteins (**Supplementary Figure S2(iii)**), although terms related to transcriptional regulation were significant in the transcriptomic analysis specially amongst down-regulated DEGs in SN GVs (**Figure S1(v)C**). The general lack of correlation between transcript and protein changes (apart from a few notable exceptions) we observed (**Figure 4E**) is concordant with observations made in *Padi6*, *Tle6* and *Uhrf1* knockout oocytes (Refs 3, 23). Accordingly, we do not think we are likely to identify any new insights from the 'multi-omic' analysis.

Other changes and edits made to the manuscript

A few typos have been found and corrected (identified as tracked changes).

We have altered the first **Results** subheading to “**Whole cytoplasm localisation of NALP5 in germinal vesicle oocytes**” as the original was an incorrect statement.

Because of the addition of Figure 1 panel C, the original Figure 1C becomes **Figure 1D**, and the original Figure 1D becomes **Figure 1E**, and the Figure legend is updated accordingly.

Errors were found in **Supplementary Table S3**
1. **Table S3(i)**: column C heading has been corrected to ‘CpGs covered’ from ‘GpGs covered’.

2. The data in **Table S3(i)** column C was incorrect because of the wrong placement of ‘commas’ and ‘decimal points’ in the numerical values. For example:
cell C2 was written ‘4,797.94’ but should have been ‘4797935’
cell C3 was written ’11,506.12’ but should have been ‘11506116’
All values in column C have now been corrected.

3. **Table S3(ii)** and **(iii)** report the methylation values of imprinted gDMRs. In the original tables, values were reported for the individual 100-CpG tiles overlapping with the gDMRs. We have changed this to report methylation values for each entire gDMR, which corresponds to how the data are presented in Figures 7 C(v) and D.

First revision: MHR-25-0090.R1

Reviewer 2
The authors addressed all the points and the manuscript is now suitable for publication in its present form.

Associate Editor
As the reviewer mentioned the authors have addressed the comments and I support the manuscript to be published.

Editor-in Chief
The Authors performed a comprehensive revision that settled all previous critiques. We thank the Authors for explaining in such detail what they did (rebuttal) and congratulate the Authors on the positive outcome.
